# Identification and validation of a novel phagocytosis regulators-related signature with potential prognostic and immunotherapeutic value in patients with lung adenocarcinoma

**DOI:** 10.3389/fonc.2022.988332

**Published:** 2022-11-02

**Authors:** Jingyang Li, Qinyun Du, Jiayi Sun, Li Xiang, Shaohui Wang

**Affiliations:** ^1^ State Key Laboratory of Southwestern Chinese Medicine Resources, School of Pharmacy, Chengdu University of Traditional Chinese Medicine, Chengdu, China; ^2^ State Key Laboratory of Southwestern Chinese Medicine Resources, Innovative Institute of Chinese Medicine and Pharmacy, Chengdu University of Traditional Chinese Medicine, Chengdu, China; ^3^ State Key Laboratory of Southwestern Chinese Medicine Resources, School of Ethnic Medicine, Chengdu University of Traditional Chinese Medicine, Chengdu, China

**Keywords:** lung adenocarcinoma, phagocytosis regulators, prognostic value, immunotherapeutic value, risk score

## Abstract

**Background:**

Lung adenocarcinoma (LUAD) is a malignant tumor that seriously affects the prognosis of patients. Tumor-associated macrophages (TAMs) play a vital role in the tumor microenvironment and can be used as a potential target for tumor therapy, and phagocytosis regulators (PRs) are particularly important in this process. However, the PRs-related signature that can predict the potential prognostic and immunotherapeutic value in patients with LUAD has not been discovered.

**Methods:**

In this study, we mainly analyzed the effect of phagocytosis regulators on the prognosis of LUAD, and based on multiple screening analyses including differential analysis, univariate Cox analysis, and Lasso analysis, we constructed a prognostic risk model consisting of five genes. To verify the stability of the model, survival analysis and ROC curve verification were carried out through multiple data sets. In addition, we also combined many factors, such as immune infiltrating cells, clinical grouping characteristics, immune examination sites, pro-inflammatory factors, and other factors as well as *in vitro* cell experiments and clinical tissue samples for further validation analysis.

**Results:**

After identifying 29 differentially expressed PRs in LUAD samples, we further constructed a prognostic model consisting of five prognostic signatures (FURIN, KIF23, SASH3, GNPNAT1, and ITGAL). Further survival analysis tests, ROC verification, as well as univariate and multivariate Cox regression analysis showed that the risk score of the model could well predict the prognosis of LUAD patients and could be used as an independent prognostic factor. In addition, we further found that these phagocytic regulators-related signatures were closely related to the immune microenvironment and immunotherapy in LUAD patients, and could well predict the efficacy of immunotherapy in patients. *In vitro* cell experiments and Immunohistochemistry of clinical tissues showed that the expressions of FURIN, KIF23, SASH3, GNPNAT1 and ITGAL in normal lung cells/tissues and LUAD cells/tissues were consistent with bioinformatics results, and 3 of them had significant differences.

**Conclusion:**

Our study identified a novel PRs-related signature that has potential application value in predicting the prognosis of LUAD patients and predicting the efficacy of immunotherapy. This provides a new basis for the prognosis assessment of LUAD patients and provides a novel target for immunotherapy of LUAD patients.

## 1 Introduction

Globally, lung cancer was the leading cause of cancer-related deaths in 2020 and accounted for approximately 11.4% of all cancers and 18% of deaths (1). Non-small cell lung cancer (NSCLC) is the most common subtype of lung cancer, which consists of two main histological types: lung adenocarcinoma (LUAD), and lung squamous cell carcinoma (LUSC) (2). Despite major advances in surgery, new chemotherapy, and radiation treatments, survival rates for lung cancer remain grim. The 5-year survival rate for advanced NSCLC is less than 3% (3). The low survival rate of lung cancer can be attributed to its high rate of metastasis and recurrence, as well as severe drug resistance (4). In particular, LUAD is more prone to distant hematogenous, hematogenous, and lymphatic metastasis than LUSC, and the prognosis is relatively poor. Therefore, the discovery of new potential prognostic biomarkers and therapies is imperative for patients with LUAD.

The tumor microenvironment has a profound influence on the development, therapeutic effect, and prognosis of a tumor (5, 6). Tumor-associated macrophages (TAMs) are the main immune cells in the tumor microenvironment (6). Macrophages are the main component of leukocyte infiltration, and there are different numbers of macrophages in all tumors, studies have shown that tumor-associated macrophages can be used as tumor therapeutic targets (7). Macrophages are a key driver of tumor inflammation, and TAMs contribute to tumor progression at various levels, including promoting genetic instability, culturing cancer stem cells, paving the way for metastasis, and taming protective adaptive immunity (8, 9). TAMs express triggers of the checkpoint that the regulation of T cell, and is a target of the checkpoint blocking immunotherapy. Macrophage-centered therapies include strategies to prevent tumor recruitment and survival, induce extracellular killing or phagocytosis of cancer cells, and so on (8, 10). Therefore, identification and characterization of phagocytosis regulators (PRs) are particularly important to elucidate the mechanisms of phagocytosis in LUAD.

The rapid development of bioinformatics provides us with an effective way to explore the key biomarkers and molecular mechanisms of the occurrence and development of diseases (such as cancers, diabetic nephropathy, etc.) (11–13). In this study, we aimed to characterize the influence of PRs on the occurrence and development of LUAD by using the PRs identified in two genome-wide CRISPR articles (14, 15), and constructed the PRs signature with potential prognostic value. Further, we explored the interaction between the signature and macrophages, and predicted its prognosis and immunotherapeutic effect in LUAD patients.

## 2 Materials and methods

### 2.1 Data collection

The genome sequencing data, clinical data, and survival information of TCGA-LUAD were downloaded from the XENA database (https://xenabrowser.net/datapages). Among them, there were 582 intersection samples of transcriptome data (standardized count data were selected due to differences) and clinical data, 526 cancer samples, 59 control samples, and 572 intersection samples combined with survival information. The GSE68465, GSE31210, and GSE135222 expression profiling datasets of human LUAD were downloaded from the GEO database (https://www.ncbi.nlm.nih.gov/geo/). The basic clinical data of the above patients with LUAD were shown in **Supplementary Figure S1**.

### 2.2 Correlation analysis and group comparison analysis

The ssGSEA (single sample gene set enrichment analysis) analysis method was used to obtain the macrophage enrichment score, and further conducted a Pearson correlation analysis combined with the expression of PRs in samples. Then, according to the median expression value of phagocytic regulatory factors, we grouped them into high and low expression groups, and plotted the corresponding boxplot.

### 2.3 Enrichment analysis

ClusterProfiler package was used for GO and KEGG analysis, and its filtering parameter was pAdjustMethod =‘none’, pvalueCutoff = 0.05, qvalueCutoff = l.

### 2.4 Analysis of gene expression differences

Differences in gene expression analysis by using DEseq2 in the R package, |log2FC| ≥0.8 and padj<0.05 was used as the screening standard.

### 2.5 Screening of prognostic-related factors and construction and evaluation of prognostic risk scoring model

According to the influence of the expression level of differential expression factors on the survival time of patients, the univariate Cox regression model was used to identify the prognostic factors, and the threshold was selected as p<0.01. In addition, Lasso-logistic regression was used to remove redundant factors and further screen for prognostic factors. For the screened prognostic factors, the risk score was calculated according to the risk ratio regression coefficient in the multi-factor Cox regression model and its expression level, and finally, the prognostic risk Score model was constructed. Then, the samples were grouped according to the median risk score, and the correlation between risk score and patient survival time was analyzed by log-rank test. The ROC curve was drawn by R-package timeROC to evaluate the prognostic efficacy of the risk model. At the same time, GEO data were used for verification analysis.

In detail, for the Cox analysis, the coxph function of the survival package was used for the Cox analysis of samples and corresponding genes. Cox analysis can be divided into univariate and multivariate Cox regression analyses. In univariate Cox regression analysis, target genes were treated as independent factors affecting prognosis for regression analysis, and the risk score and significant degree of each gene were calculated. However, in multivariate Cox regression analysis, target genes are treated as cofactors associated with each other. By analyzing the multivariate Cox regression coefficient of each gene, the sum of the product of the multivariate Cox regression coefficient and the expression level of the corresponding gene was used as the risk value to measure the risk degree of the sample. Cox analysis was performed under the default parameters of the coxph function, and the significant degree was p<0.05 as the standard.

Risk score_i_ =


(1) $$\sum_{j=1}^{n}C\text{j}*\exp ij$$

This formula calculated the Risk score value of the ith sample. Where C_j_ was the regression coefficient of the jth prognostic factor in the Cox regression model, exp_ij_ was the expression of the jth prognostic factor in the ith sample.For Lasso-logistic regression analysis, we used the glmnet function of the glmnet package to perform Lasso analysis on samples and corresponding genes. The parameters used in the lasso analysis were alpha=1, nlamba=100, and the significance degree was P<0.05 as the standard.

### 2.6 Immunoinfiltration analysis

Tumor immune invasion analysis was performed based on TCGA gene expression data by using the cibersort package to analyze the proportion of tumor immune cells in samples with cibersort default parameters. The results of immune infiltration calculated by the TIMER algorithm and XCELL algorithm were obtained by TIMER2.0 (http://timer.cistrome.org/) online analysis website.

### 2.7 Evaluation of immune score and gene score

The immunological scores and genetic scores were performed using the R package ESTIMATE under default parameters.

### 2.8 Cell culture

The human lung cancer cell lines (H1299, A549) were obtained from the Shanghai Zhong Qiao Xin Zhou Biotechnology Co., Ltd. The normal lung cell lines (BEAS-2B) were purchased from Hunan Fenghui Biotechnology Co., Ltd. H1299 and A549 were maintained in RIPM-1640 medium supplemented with 10% fetal bovine serum and antibiotics. BEAS-2B was maintained in Dulbecco’s modified Eagle’s medium (DMEM) supplemented with 10% fetal bovine serum and antibiotics. All cells were incubated in 5% CO^2^ at 37°C.

### 2.9 Quantitative real-time PCR and immunohistochemistry

The total RNA was extracted with RNA isolation Kit V2 (Vazyme, RC112) according to the product protocol. A reverse transcription reaction was carried out to acquire cDNA to prepare for the quantitative real-time PCR with the ABScript III RT Master Mix (ABclonal, RK20428). qPCR was cycled with the quantitative real-time gene amplification instrument (Jena qTower 3g) using 2X SYBR green Fast qPCR Mix (ABclonal, RK21205). Primers for GNPNAT1, SASH3, KIF23, FURIN, and ITGAL (Tsingke Biotechnology Co., Ltd) were listed in **Supplementary Table S1**. GADPH was used as endogenous control and further analyzed by the 2ΔddCt method. The amplification efficiency was assessed by the standard curve. The experiment was repeated three times. Further, we analyzed the immunohistochemical results of FURIN, KIF23, SASH3, GNPNAT1 and ITGAL by screening the HPA database (https://www.proteinatlas.org/).

## 3 Results

### 3.1 PRs could regulate macrophage phagocytosis and participate in the occurrence and development of lung adenocarcinoma

#### 3.1.1 PRs could regulate the phagocytosis of macrophages

**Figure 1:**
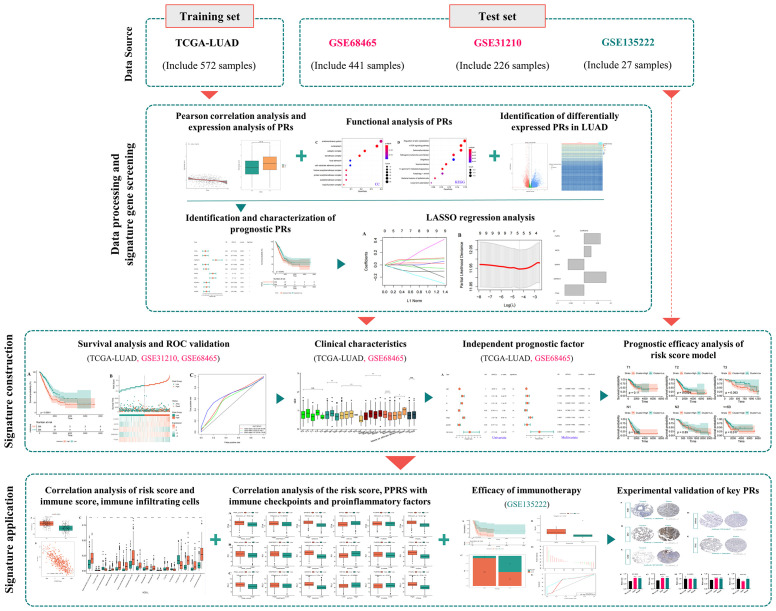
The analysis flowchart for identification and validation of a novel phagocytosis regulators-related signature with potential prognostic and immunotherapeutic value in LUAD.

**Figure 2:**
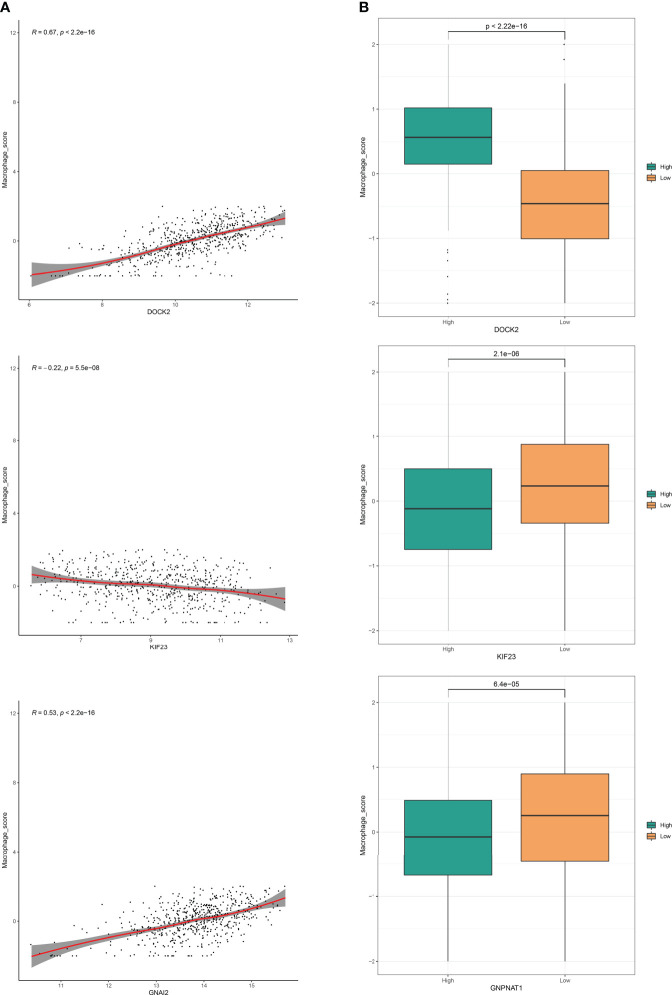
Phagocytosis regulators could regulate the phagocytosis of macrophages. **(A)** Correlation analysis of phagocytosis regulators expression level and macrophage enrichment score in LUAD samples. **(B)** Grouping comparison of phagocytosis regulators and macrophage enrichment scores.

The analysis workflow is shown in **Figure 1**. We first obtained 183 PRs (**Supplementary Table S2**) from the two genome-wide CRISPR articles (14, 15), among which 178 genes existed in the TCGA dataset we used. Pearson correlation analysis showed that 118 genes had significant differences among these PRs, among which 69 were positively correlated and 49 were negatively correlated (**Supplementary Figure S2**). The group comparison result showed that a total of 92 PRs were significantly different, among which 43 PRs were significantly enriched in the high expression group. On the contrary, 49 PRs were significantly enriched in the low expression group (**Supplementary Figure S3**). Together, these results demonstrated that the most PRs could regulate the phagocytosis of macrophages and participate in the development of LUAD. **Figure 2** showed the correlations and boxplots of partial PRs.

#### 3.1.2 Functional analysis of PRs

**Figure 3:**
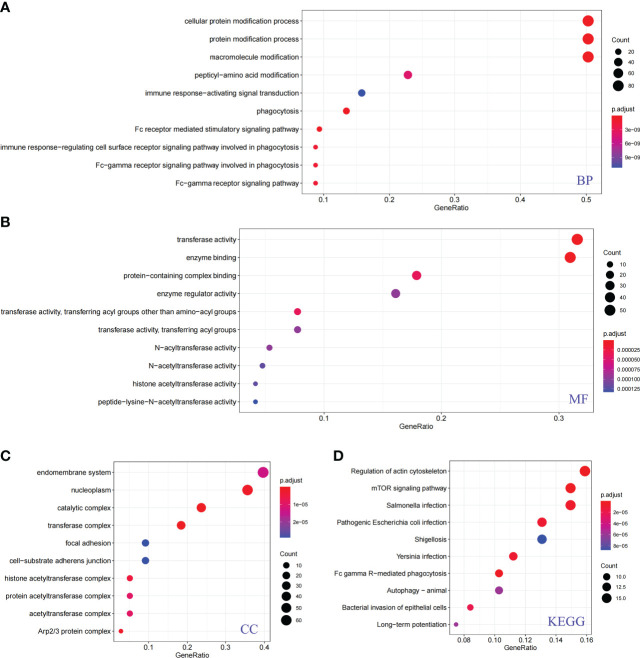
Functional analysis of phagocytosis regulators. **(A)** Biological process. **(B)** Molecular function. **(C)** Cellular component. **(D)** KEGG pathway.

To fully explore the potential functions and pathways of PRs, we further performed GO and KEGG enrichment analyses. The ClusterProfiler package was used for GO and KEGG analysis of PRs, and the results were shown in **Figure 3**. The results showed that these phagocytic regulatory factors were significantly different in cellular protein modification process (**Figure 3A**), transferase activity (**Figure 3B**), enzyme binding (**Figure 3B**), endomembrane system (**Figure 3C**), regulation of actin cytoskeleton (**Figure 3D**) and mTOR (**Figure 3D**) related cell functions and signaling pathways.

#### 3.1.3 Identification of differentially expressed PRs in LUAD

**Figure 4:**
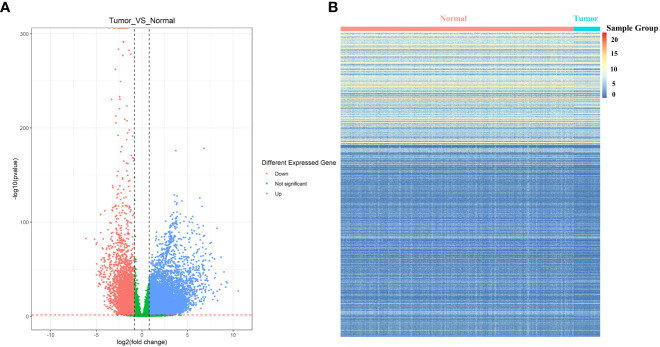
Identification of differentially expressed phagocytosis regulators in LUAD. **(A)** Volcano map of differentially expressed genes. **(B)** Expression heat map of differentially expressed genes. The normal sample shown here has more dark color changes than the tumor sample.

In order to further identify the differentially expressed PRs in LUAD, the DEseq2 package was used for differential expression analysis of the TCGA database, and the volcano maps and expression heat maps (R package pheatmap) were drawn. Through the analysis of the volcano map (**Figure 4A**) and heat map (**Figure 4B**), we found that among all differentially expressed genes in LUAD, a total of 29 PRs were present, including 13 up-regulated genes and 16 down-regulated genes.

### 3.2 Identification and characterization of prognostic PRs

**Figure 5:**
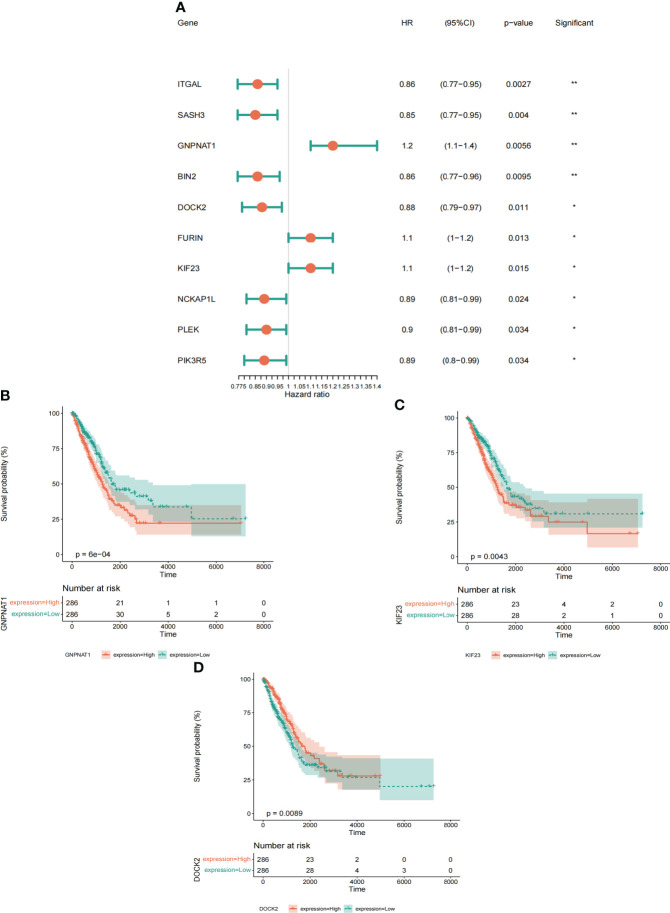
Identification and characterization of prognostic phagocytosis regulators. **(A)** Univariate Cox regression analysis of prognostic phagocytosis regulators in LUAD patients. **(B-D)** Kaplan-meier curves of phagocytosis regulators (Including GMPNAT1, KIF23 and DOCK2). *p < 0.05, **p < 0.01.

To determine whether the differential expression of PRs is related to the prognosis of LUAD patients, based on the expression data and survival information of LUAD in TCGA, we further used differentially expressed PRs for univariate Cox screening. Finally, we obtained 10 prognostic PRs (**Figure 5A**), among which GMPNAT1, KIF23 and DOCK2 genes were independent prognostic genes (**Figures 5B-D**, **Supplementary Figure S4**).

### 3.3 Construction of prognostic PRs-related signature model

**Figure 6:**
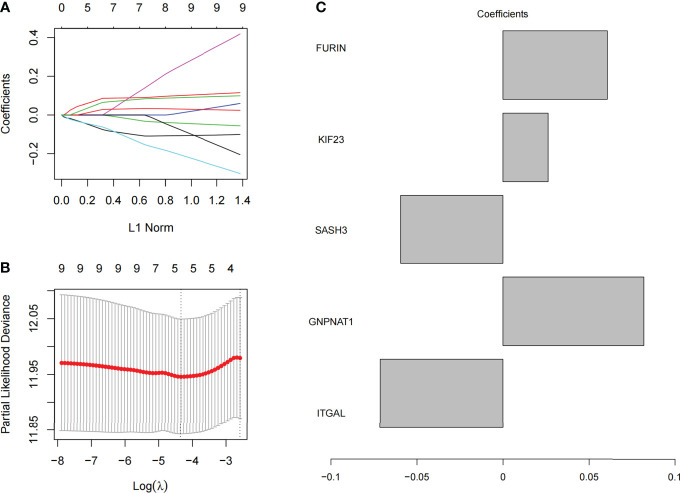
Model construction of phagocytosis regulators signature. **(A)** The coefficient of 10 prognostic phagocytosis regulators signature. **(B)** the partial likelihood deviance of prognostic phagocytosis regulators signature. **(C)** The coefficient display of the 5 phagocytosis regulators signature in the optimal combination model.

We selected 10 prognostic PRs as research objects, and further reduced the number of target gene sets by Lasso analysis. As a result, we constructed a signature model with 5 significant prognostic PRs through Lasso analysis (**Figures 6A, B** **)**, and the formula of the optimal model is as follows: risk score = (0.06062) * FURIN + (0.02622) * KIF23 + (-0.05976) * SASH3 + (0.08207) * GNPNAT1 + (-0.07163) * ITGAL (**Figure 6C**).

### 3.4 Risk score was correlated with the prognosis and clinical features of LUAD patients

#### 3.4.1 Risk score could predict the outcome of LUAD patients

**Figure 7:**
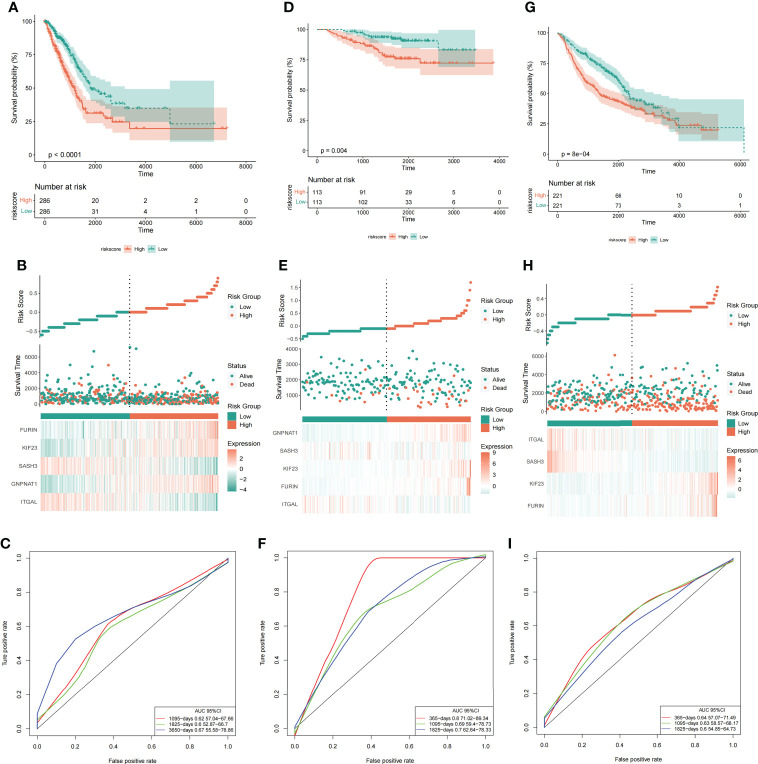
Risk score could predict the outcome of LUAD patients. Survival analysis **(A, D, G)**, risk score and grouping of samples **(B, E, H)**, and ROC curve **(C, F, I)** in the training set (TCGA) and two validation sets (GSE31210 and GSE68465). From left to right are the TCGA, GSE31210 and GSE68465 datasets respectively.

To test whether the final model is stable, Further, we divided the samples into high-low risk groups based on the median of risk score, and conducted a survival analysis test and ROC verification in the training set (TCGA) (**Figures 7A-C** **)** and two validation sets: GSE31210 (**Figures 7D-F** **)** and GSE68465 (**Figures 7G-I** **)** respectively to evaluate the prognostic efficacy of risk score in LUAD patients. Surprisingly, our results showed that the survival analysis (**Figures 7A, D, G** **)** and ROC curve (**Figures 7C, F, I** **)** of the three data sets proved to be meaningful, and we preliminarily believed that the risk prediction score had a certain ability to predict risk.

#### 3.4.2 Risk score was correlated with clinical characteristics of LUAD patients

**Figure 8:**
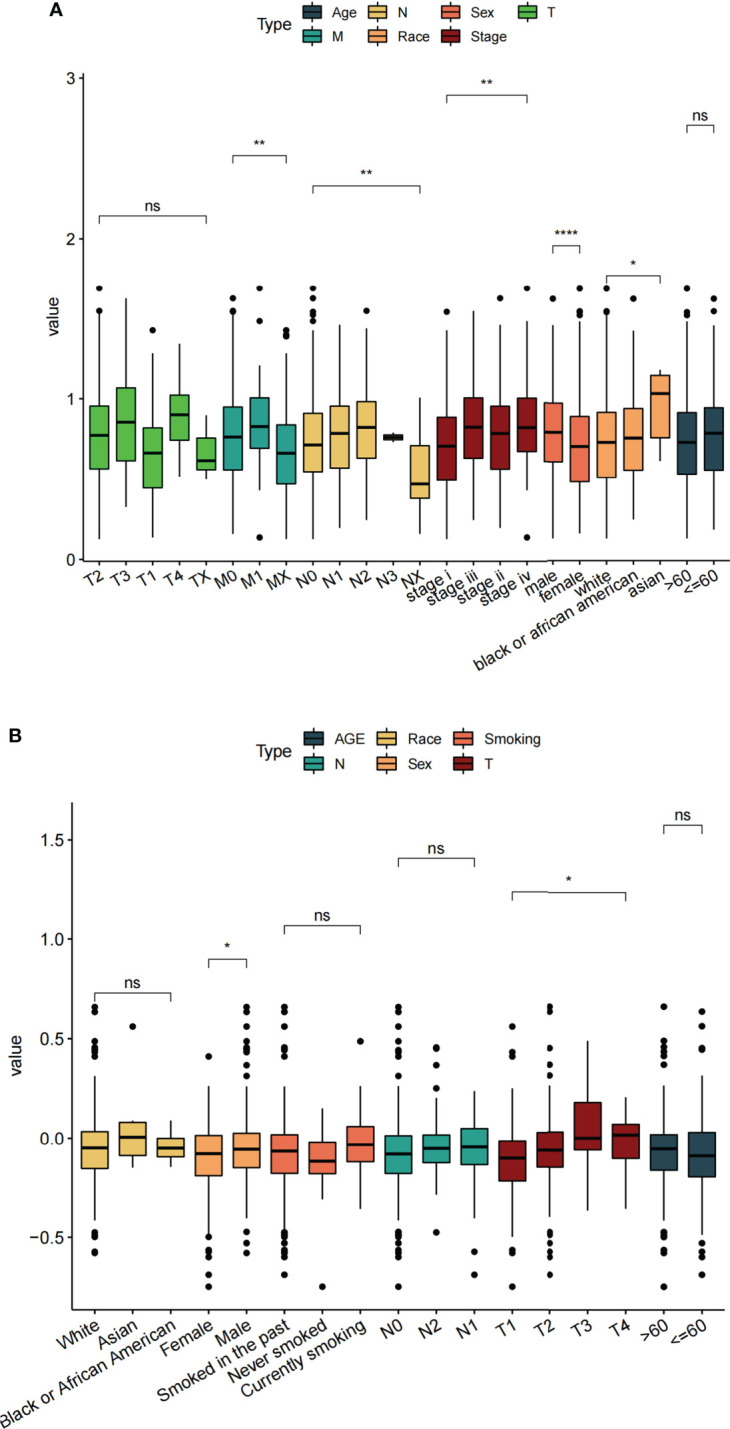
Correlation between different clinical features and risk score. **(A)** TCGA datasets. **(B)** GSE68465 datasets. ns: *p >* 0.05, *p < 0.05, **p < 0.01, ****p < 0.0001.

Based on clinical information from the TCGA dataset and the validation set (GSE68465), we assessed the relationship between risk score and clinical characteristics (including age, sex, TNM stage, race, etc.). The results showed that in the TCGA data set, the clinical parameters M stage, N stage, sex, and race were significantly correlated with risk score (**Figure 8A**). While in the GEO data set, sex and T stage were significantly correlated with risk score (**Figure 8B**). By analyzing the correlation between risk score and clinical characteristics of LUAD patients, we found that the sex was most associated with risk score.

#### 3.4.3 Risk score could be an independent prognostic factor of LUAD patients

**Figure 9:**
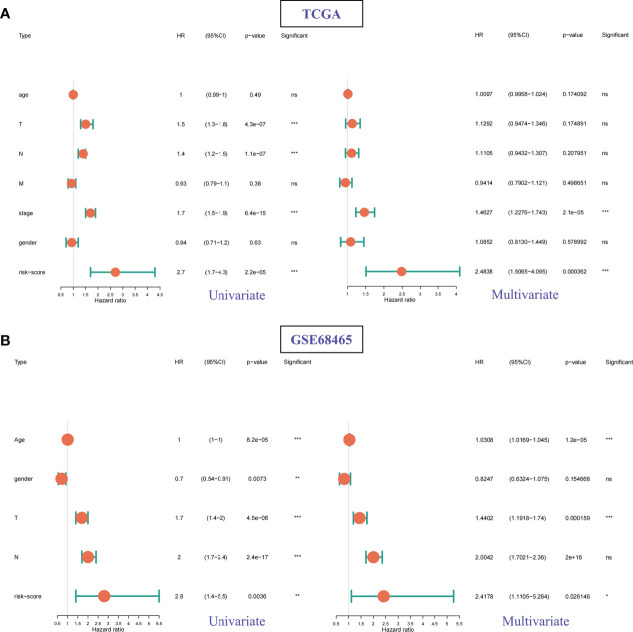
Univariate Cox and multivariate Cox regression analysis for different clinical features and risk scores. **(A)** TCGA datasets. **(B)** GSE68465 datasets. ns: p > 0.05, *p < 0.05, **p < 0.01, ***p < 0.001.

According to the clinical information of TCGA (**Figure 9A**) and GSE68465 (**Figure 9B**) data sets, univariate Cox analysis, and multivariate Cox analysis were performed on the two data sets respectively. The results showed that the *P* values of risk scores of both univariate Cox analysis and multivariate Cox analysis were less than 0.05 in these two data sets (**Figure 9**). Therefore, we can consider the risk score of this model as an independent prognostic factor for LUAD patients.

#### 3.4.4 Prognostic efficacy analysis of risk score model

**Figure 10:**
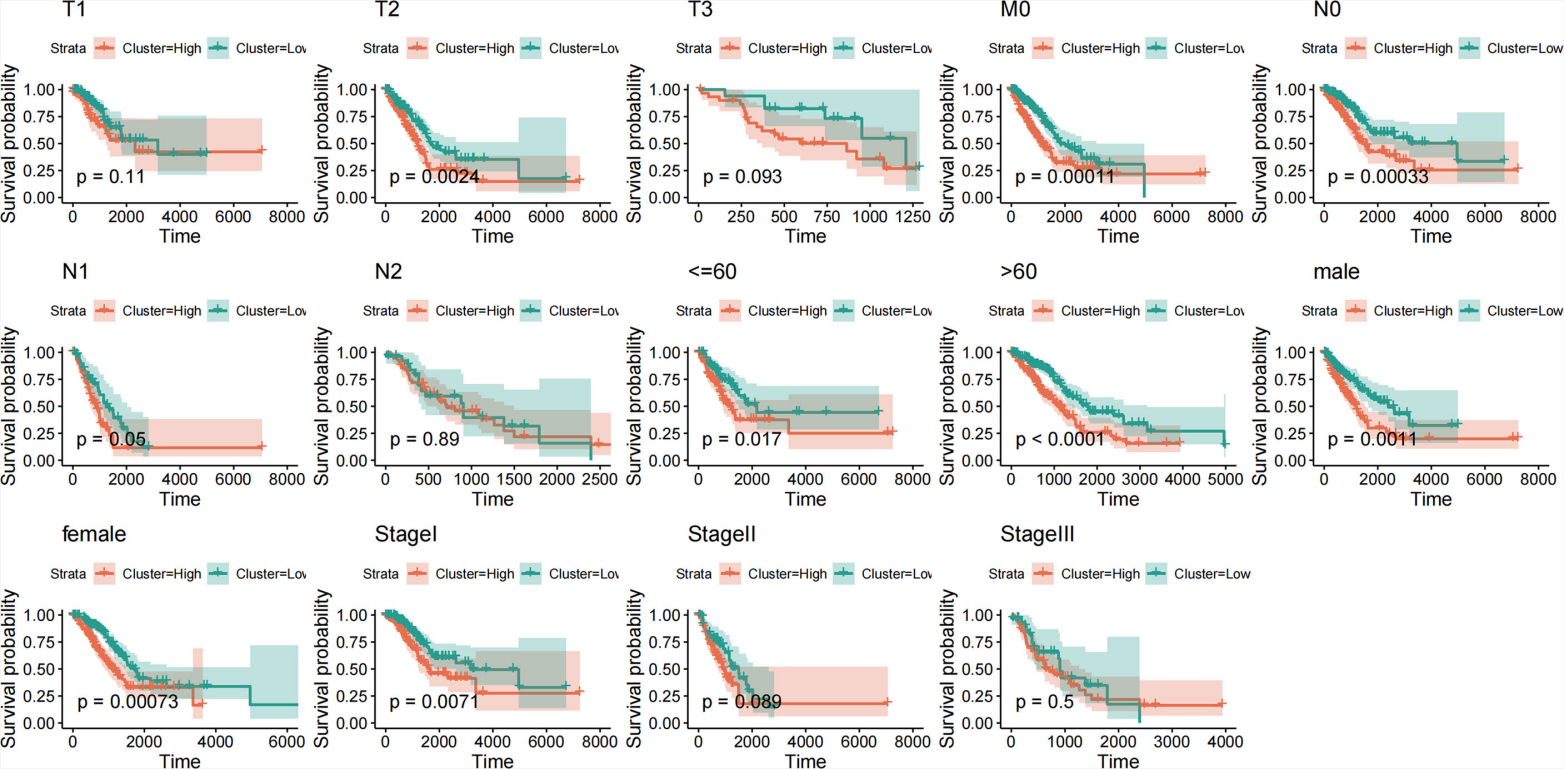
Prognostic efficacy and stability assessment of the risk score model.

To evaluate the effectiveness and stability of the risk scoring model, we further analyzed the TCGA dataset. By extracting the clinical characteristics of LUAD samples from this dataset, including age, sex, stage, and TNM stage. Based on the above clinical information, the above risk scoring model was used to group the risk prediction of LUAD samples and compare the prognosis between groups. It is not difficult to find that risk score model with various clinical information, such as age, gender, sample stage and sample TMN stage, have significant inter-group prognostic differences in these clinical characteristics (**Figure 10**). These clinical features are also indicative clinical features of LUAD. The above results show that the prediction efficiency and stability of this model are high.

### 3.5 PRs signature was related to the immune microenvironment and immunotherapy of LUAD patients

#### 3.5.1 Correlation analysis of risk score and immune score, immune infiltrating cells

**Figure 11:**
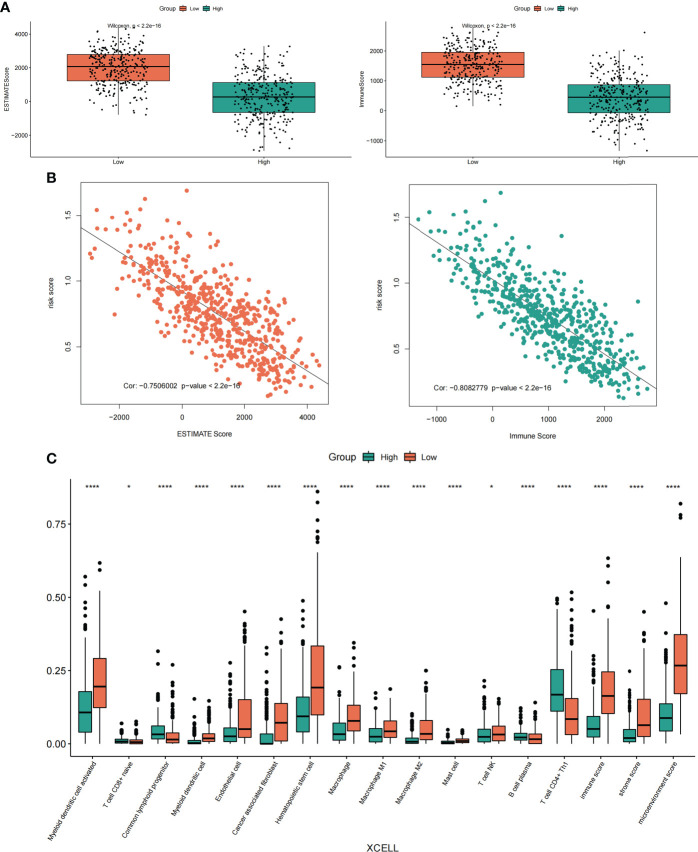
Correlation analysis of risk score, immune score and immune infiltrating cells. **(A)** Differential expression of ImmuneScore and EstimateScore scores in the high-low risk groups of LUAD samples. **(B)** Correlation analysis between ImmuneScore and EstimateScore scores and LUAD sample risk score. **(C)** The expression differences of different immune cell infiltrates between the high and low-risk groups were analyzed by the XCELL algorithm. *p < 0.05, ****p < 0.0001.

To determine the relationship between risk score and the tumor immune microenvironment, we first used the ESTIMATE method to calculate the ImmuneScore and EstimateScore scores of LUAD samples, and then we combined with the risk scores of LUAD samples to analyze the expression difference between the two types of scores (ImmuneScore and EstimateScore) in the case of high and low-risk groups (**Figure 11A**), as well as the correlation between the two types of scores and risk scores (**Figure 11B**). Excitingly, we found that the enrichment scores of these two types of immunity in the high-risk group were significantly lower than those in the low-risk group. moreover, correlation analysis showed that the two types of immunity scores were significantly negatively correlated with the risk score.

In addition, we also used TIMER2.0 (http://timer.cistrome.org/) online analysis website to obtain the immune infiltration of LUAD samples calculated by the TIMER algorithm and XCELL algorithm. Meanwhile, the Cibersort algorithm was also used to calculate the immune infiltration of LUAD samples. And the results showed that the immune infiltration scores obtained by the XECLL algorithm were different among groups under the high and low-risk group of LUAD samples, including macrophages, myeloid dendritic cell, B cell, T cell CD4^+^, T cell CD8^+^, etc. (**Figure 11C**). Similarly, the two other algorithms (TIMER algorithm and Cibersort algorithm) also showed similar results (**Supplementary Figure S5**, **Supplementary Figure S6**). In conclusion, our results showed that there was a strong correlation between these risk scores and immune scores and immune infiltrating cells, especially in macrophages.

#### 3.5.2 Correlation analysis of the risk score, PPRS with immune checkpoints and proinflammatory factors

**Figure 12:**
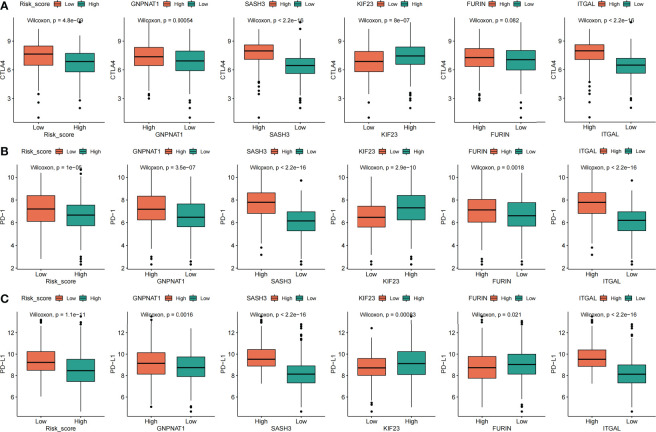
Correlation analysis between risk score, five prognostic phagocytosis regulators signatures and immune checkpoints. **(A)** CTLA4. **(B)** PD-1. **(C)** PD-L1.

According to the risk score of LUAD samples and the high and low expression of the five prognostic PRs that constitute the model, and LUAD patients were divided into two subgroups, the high expression group and the low expression group. Subsequently, the correlation analysis of the risk score and the five prognostic phagocytosis regulators signature (PPRS) with immune checkpoints (PD-1, PD-L1, CTLA4) and proinflammatory factors (IL-1α, IL-1β, IL-6, IL-8 and IL-18) was conducted. we found that CTLA4 was not significantly different in the high and low groups of FURIN, while there was a significant difference between the groups with high and low expression of other factors (**Figure 12A**). In addition, the PD-1 expression level was negatively correlated with a risk score, and positively correlated with GNPNAT1, SASH3, KIF23, FURIN, and ITGAL (**Figure 12B**). The PD-L1 expression level was negatively correlated with risk score and FURIN, and positively correlated with GNPNAT1, SASH3, KIF23 and ITGAL (**Figure 12C**). By analyzing the correlation between major pro-inflammatory factors and risk score and the five PPRS, we found that the most pro-inflammatory factors (IL-1α, IL-1β, IL-6, IL-8 and IL-18) were significantly correlated with the risk score and the five PPRS (**Supplementary Figure S7**).

#### 3.5.3 The model could predict the efficacy of immunotherapy in LUAD patients

**Figure 13:**
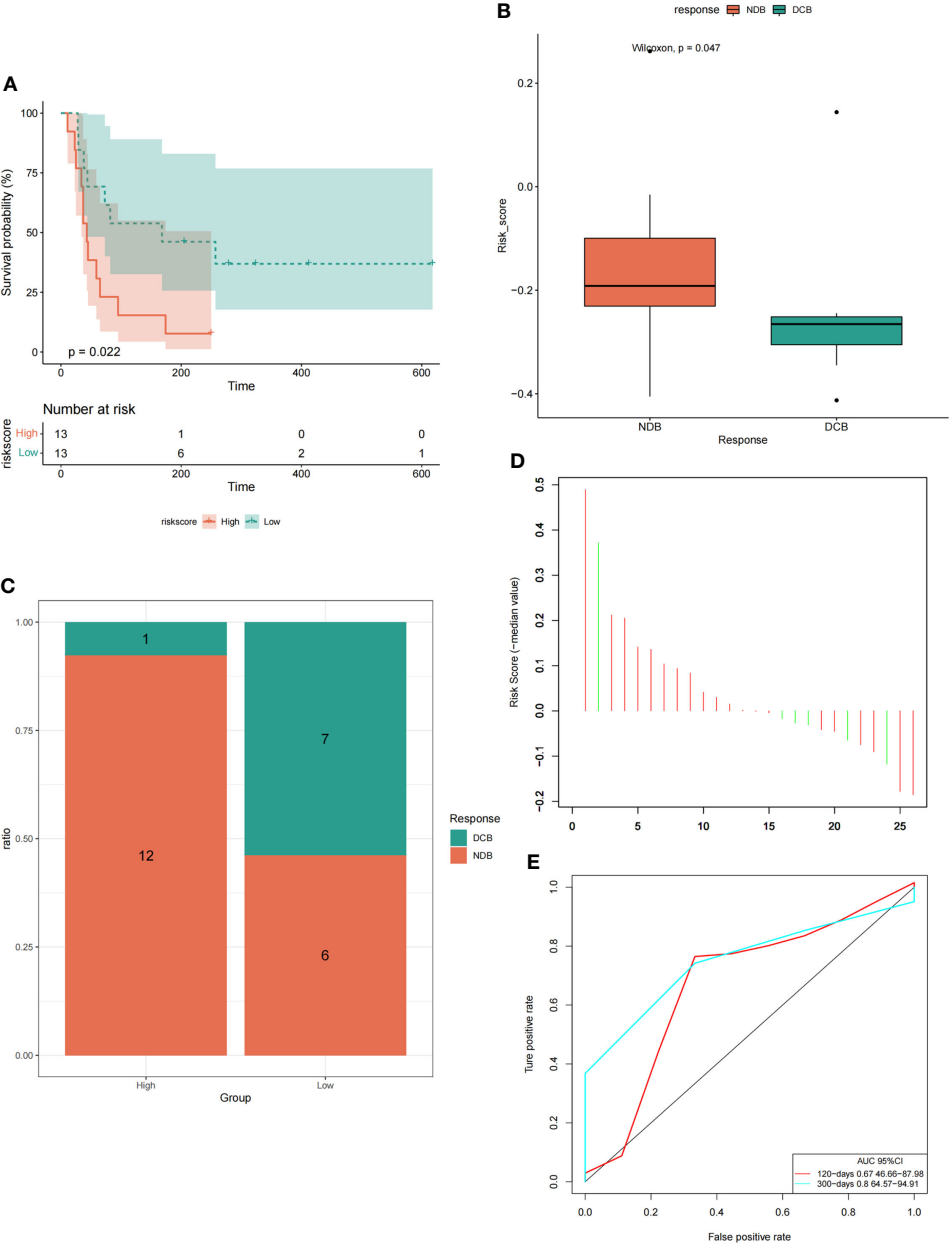
The model could predict the efficacy of immunotherapy in LUAD patients. **(A)** Survival curve analysis under high and low-risk groups. **(B)** Analysis of intergroup differences in risk scores under two immunotherapy responses. **(C)** The proportion of two immunotherapy responses in the high-low risk group. **(D)** The risk score of the LUAD samples. **(E)** ROC verification curve of the model.

Model prediction score was performed for GSE135222 dataset containing immunotherapy response, and the survival analysis curve and ROC verification curve were drawn according to the PFS information of the dataset. In addition, the correlation bars were plotted based on immunotherapy responses (DCB: durable clinical benefit, including LUAD patients with CR, PR and SD>6 months; NDB: non-durable clinical benefit, including LUAD patients with SD ≤ 6 months and PD) in the data set, and the difference of risk score under different immunotherapy response subgroups were analyzed. We found that in the GSE135222 data set, the high-risk group had a worse prognosis than the low-risk group (**Figure 13A**), which was consistent with the previous results. The risk score was higher in the NDB immunotherapy response than in the DCB immunotherapy response (**Figure 13B**), and NDB was the largest proportion in the high-risk group (**Figure 13C**). Further ROC curve verification showed that the areas under the ROC curve at 120, and 300 days were 0.67 and 0.8, respectively (**Figures 13D, E** **)**.

### 3.6 Experimental validation of key PRs

**Figure 14:**
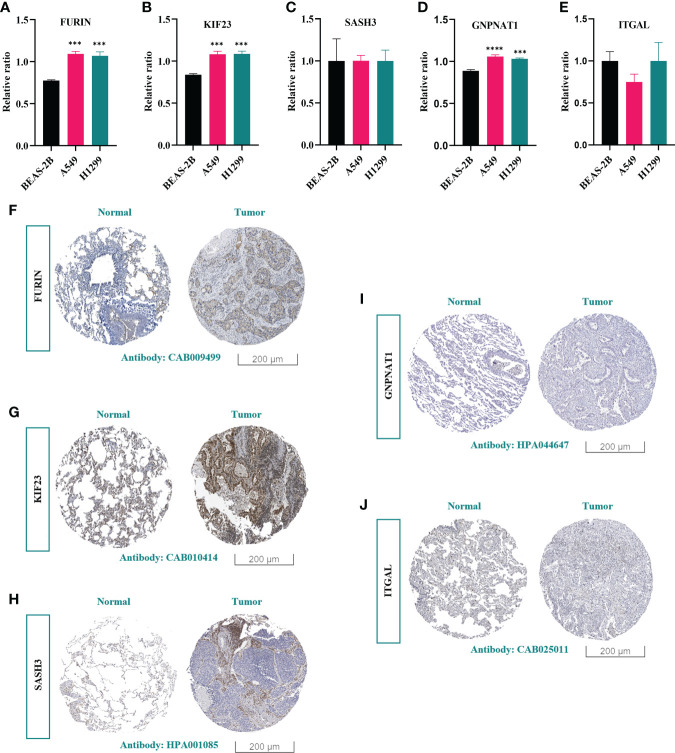
Experimental validation of key PRs. **(A-E)** The expression levels of 5 prognostic phagocytosis regulators in the model in BEAS-2B, A549 and H1299 cell lines were detected by RT-qPCR. ***: *p* < 0.001, ****: *p* < 0.0001. **(F-J)** Immunohistochemical results of FURIN, KIF23, SASH3, GNPNAT1 and ITGAL.

To confirm that PRs play an important role in LUAD, we further compared their expression in normal lung epithelial cells (BEAS-2B) and different lung adenocarcinoma cell lines (A549 and H1299). The results showed that the expression of these 5 genes in the above cell lines was consistent with the expression trend in LUAD patients, and 3 of them had significant differences (**Figures 14A-E**). Further, by analyzing the results of immunohistochemistry, we could qualitatively observe significant differences in the protein expression levels of the three PRs (FURIN, KIF23 and GNPNAT1) in normal lung tissues and LUAD samples (**Figures 14F-J**).

## 4 Discussion

LUAD is one of the most serious malignant tumors threatening human life, health and quality of life in the world. At present, immunotherapy is the main frontier in the treatment of LUAD patients (16). Macrophages are key drivers of tumor inflammation, and TAM promotes tumor progression at different levels (8, 9). Phagocytosis is required for a variety of physiological functions, from pathogen defense to tissue homeostasis (14). Therefore, it is of great significance to identify and characterize phagocytosis regulatory factors and clarify their roles in LUAD prognosis and immunotherapy response for the prognosis and treatment of LUAD patients. Gene signature is a biological function model constructed from the expression data of multiple genes, which can be used to predict the prognosis and progression of many types of malignant tumors (17). Yi et al. constructed a prognostic model of 17- immune-related genes signature to predict survival and response to ICI (immune checkpoint inhibitors) in LUAD patients and the result showed that patients with a low-risk score had a better prognosis and predicted benefit from ICI treatment (18). Sun et al. established a four-gene signature named IPSLUAD (ARNTL2, ECT2, PPIA, and TUBA4A) using stability selection and Lasso COX regression. It has been proved that it has good performance in multiple LUAD queues (19). The construction of this gene signature models provides a certain reference for the prognosis and immunotherapy of LUAD patients. However, the interaction between PRs and macrophages from the perspective of phagocytosis regulators has not been explored to predict the prognosis and therapeutic effect of lung adenocarcinoma patients. In our study, we constructed a novel PRs-related signature model containing five genes. Further, we explored the interactions of these signatures with macrophages and verified their accuracy in predicting patient outcomes and therapeutic effects.

We first demonstrated that most of the 183 PRs that we identified in the literature could modulate macrophage phagocytosis in LUAD samples. Then, we analyzed the functional enrichment of these PRs and found that these phagocytic regulatory factors were mainly enriched in cellular protein modification process enzyme binding, regulation of actin cytoskeleton and mTOR, and other related functions and pathways. As pleiotropic cells, macrophages can undertake a variety of functions according to the tissue they inhabit and the state of the tissue. Studies have confirmed that mTOR plays a key role in the activation of macrophages, especially its ability to control the activation and metabolic process of macrophages (20). Further, we analyzed the differential expression of these phagocytic regulatory factors in LUAD samples, and finally, we identified a total of 29 differential expression phagocytic regulatory factors. Ten prognostic PRs were further screened by univariate Cox regression analysis, including ITGAL, SASH3, GNPNAT1, BIN2, DOCK2, FURIN, KIF23, NCKAP1L, PLEK and PIK3R5. Among them, GMPNAT1, KIF23 and DOCK2 could be used as independent prognostic genes.

Subsequently, we constructed a model with five prognostic signatures (FURIN, KIF23, SASH3, GNPNAT1, and ITGAL). Glucosamine 6-phosphateN-acetyltransferase 1 (GNPNAT1) is a key enzyme in the hexosamine biosynthetic pathway, which is abnormally expressed in tumor cells and promotes tumor progression and metastasis (21). Recently, studies have shown that abnormal expression of GNPNAT1 is related to the carcinogenesis of lung cancer. Wang et al. showed that the mRNA and protein expression levels of GNPNAT1 in lung cancer tissues were upregulated compared with normal tissues (22, 23). The expression level of GNPNAT1 is related to the clinical stage and prognosis of lung cancer. Patients with high expression of GNPNAT1 are more likely to develop advanced lung cancer, with a poor prognosis and low survival rate (24). In addition, GNPNAT1 is associated with immune infiltration in lung cancer, which has a converse correlation with infiltration of B cells, CD4^+^ T cells, and dendritic cells, all of which have antitumor effects in NSCLC (25). In particular, B cell infiltration may be one of the key reasons that caused GNPNAT1 to become a prognostic factor (26). Meanwhile, in the study of the crosstalk between GNPNAT1 and immune genes, it was found that both immunosuppressive genes (LGALS9, TGFB1, CD160, CSF1R, and CD96) and immunostimulatory genes (CD40LG, CD48, IL6R, CD27, CD40, CXCR4, LTA, CXCL12, and CD28) were negatively correlated with GNPNAT1 (27). Moreover, GNPNAT1 is associated with immune signaling and is involved in lung cancer immune evasion. In conclusion, GNPNAT1 can be used as a potential prognostic biomarker and a new immunotherapy target for lung cancer. Kinesin family member 23 (KIF23), also known as MKLP1, is a key regulator of cytokinesis, transporting organelles within cells and moving chromosomes during cell division (28). It has been observed in a variety of human malignancies and is considered as a potential tumor marker. KIF23 overexpression was recently shown in lung cancer, and was associated with a low survival rate in lung cancer patients (29). Vikberg et al. found that the elevated level of KIF23 in lung cancer may be due to the extra copy of chromosome 15, and that KIF23 plays a crucial role in the last step of mitosis, so this gene is a potential molecular marker for lung cancer treatment (30). Based on this, some studies further confirmed that RNA interference-mediated KIF23 deletion can effectively inhibit lung cancer cell growth and lung tumor formation *in vivo*, and induce apoptosis of lung cancer cell lines (31, 32). In addition, KIF23 is a potential key gene regulating hypoxia-induced tumor cell stemness in the immune microenvironment of lung tumors (33). Meanwhile, the expression of KIF23 is significantly correlated with B cell infiltration, and the interaction between KIF23 expression and B cell infiltration plays an important role in the immune response and prognosis of lung cancer, which affects the tumor microenvironment and the tumor immune effect of patients (34). Furin (PCSKC3), as an important member of proprotein processing enzyme, is overexpressed in numerous human malignancies. Furin can cut many proteins closely related to tumor development, such as Notch, Wnt, VEGF, etc., so the expression of Furin can be used as a marker of tumor progression (35). Brant et al. showed that the increased expression of Furin in lung cancer was significantly associated with a low overall survival rate (36). In view of this, several studies have shown that Furin inhibitors, such as a1-PDX, can inhibit the growth and migration of lung cancer cells, and have inhibitory effects on the growth and proliferation of xenograft human lung cancer cells (37, 38). In addition, Luo et al. found that the expression of Furin in lung cancer was significantly correlated with the expression of immunomarker genes in CD8 T cells, T cells, monocytes, TAM and dendritic cells, which confirmed the relationship between Furin expression and immune infiltration in lung cancer (39). In conclusion, Furin can be used as a promising biomarker for the judgment of immune invasion and treatment of lung cancer. SASH3 encodes a signal adapter protein that plays a role in many developmental processes, including cell growth and migration (40). Li et al. revealed that SASH3 was significantly correlated with the survival rate of lung cancer patients. Moreover, SASH3 is associated with gene regulatory sites (such as WAS and CD53) in lung cancer, which has diagnostic value for lung cancer metastasis (41). In addition, SASH3 was one of the co-expressed factors related to tumor purity, which was significantly negatively correlated with tumor purity, but positively correlated with CD8^+^ T lymphocytes and immune score (42). Therefore, SASH3 has important clinical and biological significance in the microenvironment of lung cancer. ITG subunit alpha L (ITGAL) encodes the LFA-1 (aLb2) subunit of integrin, which is highly expressed in most immune cell populations (43). The results of current research indicate that ITGAL can be identified as a prognostic indicator of lung cancer, and patients with high expression of ITGAL have a better prognosis. Pathways enriched by high expression of ITGAL are mainly related to immune cell recognition and killing of lung cancer cells (44). In particular, ITGAL is strongly positively correlated with genes related to immune surveillance and recognition, such as CD3E and CD2 of CD4^+^ T cells. The increased activity of immune cells may be the reason for the better prognosis of patients with high expression of ITGAL (45). In summary, it can be seen that these five prognostic signature genes may play an important role in LUAD. Therefore, we then carried out survival analysis and ROC curve verification through three data sets (TCGA, GSE31210, GSE68465). In addition, we also carried out clinical characteristics analysis, univariate and multivariate Cox analysis, and analyzed the prognostic efficacy of the model. The results fully prove that the model has high predictive efficiency and stability.

More and more tumor treatment strategies focus on reversing the immunosuppressive state of the tumor microenvironment, and macrophages are the key effector cells of innate immunity, whose main functions are phagocytosis and antigen presentation. Targeting TAMs can enhance tumor immune response. The development of effective phagocyte targets and the search for new innate immune checkpoints are important strategies to improve the response rate of tumor immunotherapy (46, 47). Therefore, we further analyzed the correlation between these five phagocytic factor signatures and the immune microenvironment and immunotherapy in LUAD patients. Through the above analysis, we further proved that the model composed of five PPRS could well predict the immunotherapy effect of LUAD patients. Our RT-qPCR results showed that there were significant differences in the expression of three genes (FURIN, KIF23, GNPNAT1) *in vitro* cell experiments, and this was also confirmed by the results of immunohistochemistry.

## 5 Conclusions

Taken together, our study explored and constructed a new phagocytic regulatory signature-based model from the perspective of the combination of phagocytic regulatory factors and immunity. The model has also been verified by multiple analyses of training sets and validation sets, and its stability has been confirmed by analyses of immune infiltration, immune checkpoints and pro-inflammatory factors. More importantly, the model has also been validated by immunotherapy responses. In addition, we used cell experiments and clinical tissue samples to verify the gene and protein expression in the model. In brief, the model we constructed can well predict the prognosis and immunotherapy efficacy of LUAD patients. However, our current study did not further explore the specific regulatory roles and mechanisms of these genes in LUAD. Furthermore, the stability of this model needs to be verified by more clinical samples and experiments. In the future, we can conduct further exploration from the above perspectives.

## Data availability statement

The original contributions presented in the study are included in the article/**Supplementary Material**. Further inquiries can be directed to the corresponding authors.

## Author contributions

SW and LX designed the topic; SW carried out the bioinformatics analysis; JL carried out the cell experiments and wrote the manuscript. QD and JS studied the relevant literature and reviewed the manuscript. LX reviewed and directed the manuscript. All authors contributed to the article and approved the submitted version.

## Funding

This study was supported by the National Natural Science Foundation of China (82104534, 81903902), and the Sichuan Province Science Research Special Funding Project for Postdoctoral Fellows (2022BSH044).

## Conflict of interest

The authors declare that the research was conducted in the absence of any commercial or financial relationships that could be construed as a potential conflict of interest.

## Publisher’s note

All claims expressed in this article are solely those of the authors and do not necessarily represent those of their affiliated organizations, or those of the publisher, the editors and the reviewers. Any product that may be evaluated in this article, or claim that may be made by its manufacturer, is not guaranteed or endorsed by the publisher.
