# Supplementary figures and images for: Identification and validation of a novel phagocytosis regulators-related signature with potential prognostic and immunotherapeutic value in patients with lung adenocarcinoma
Source: Front Oncol. 2022 Nov 2;12:988332. doi: 10.3389/fonc.2022.988332 (PMC9666737; doi:10.3389/fonc.2022.988332)

## Slide 1
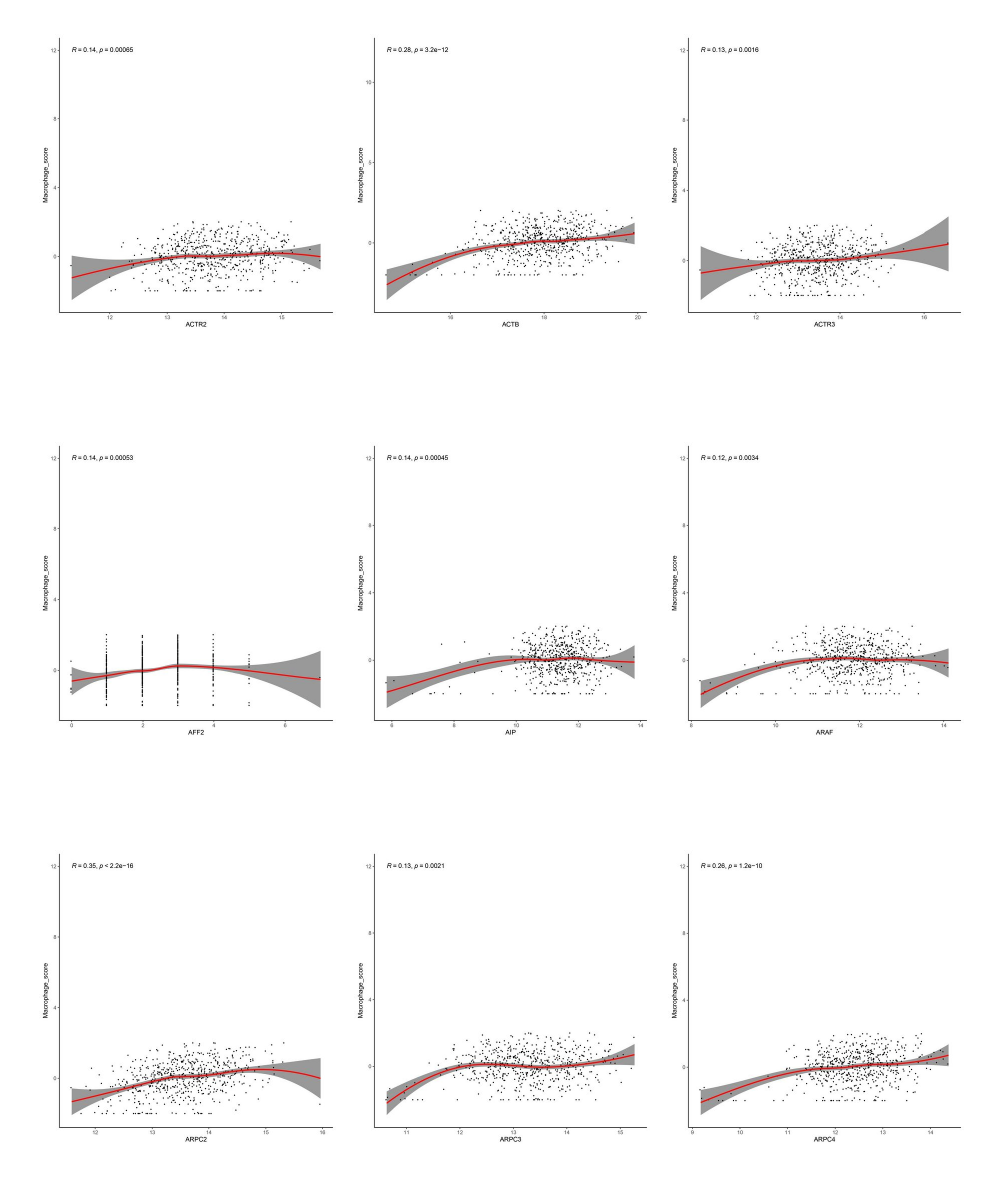

## Slide 2
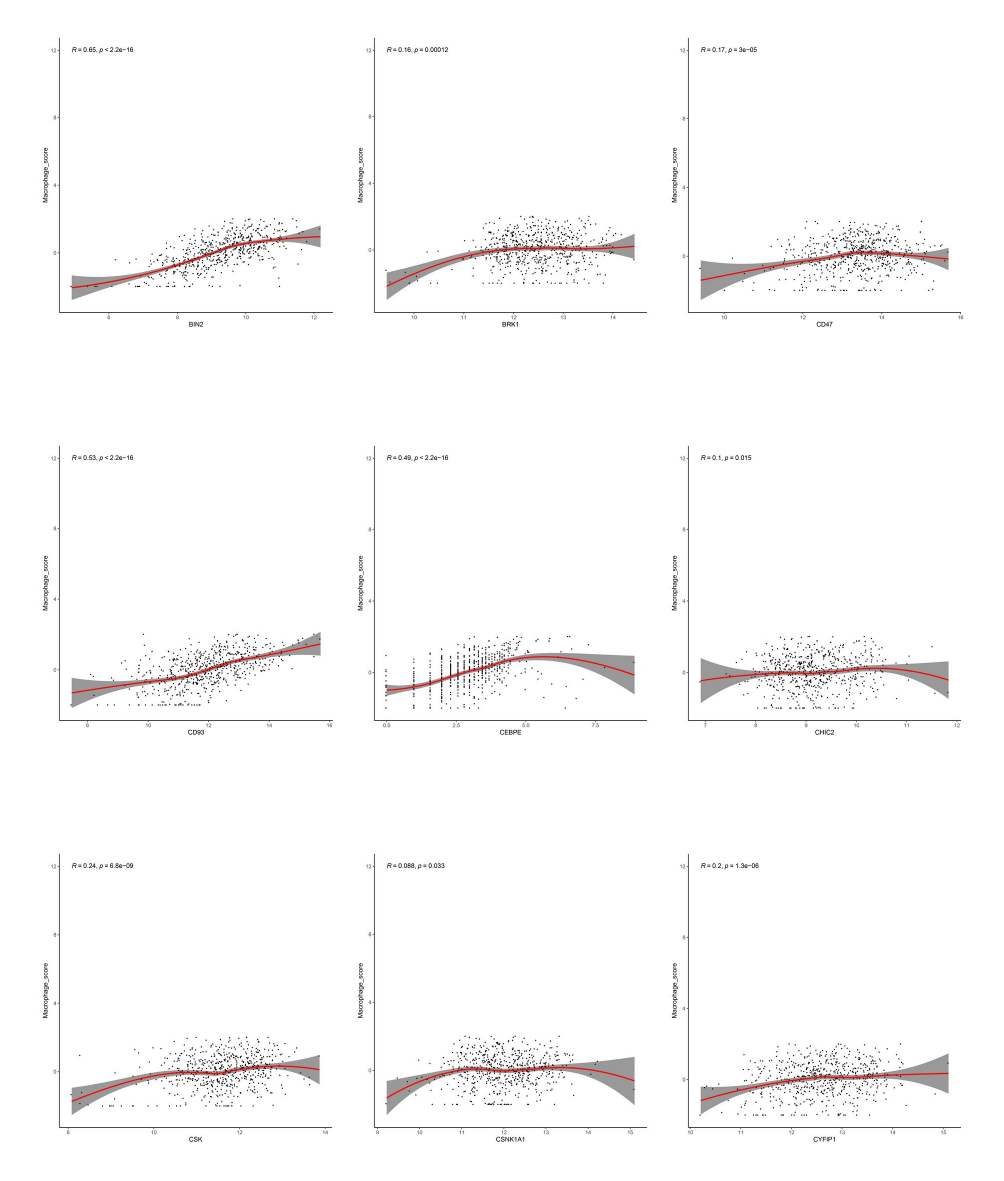

## Slide 3
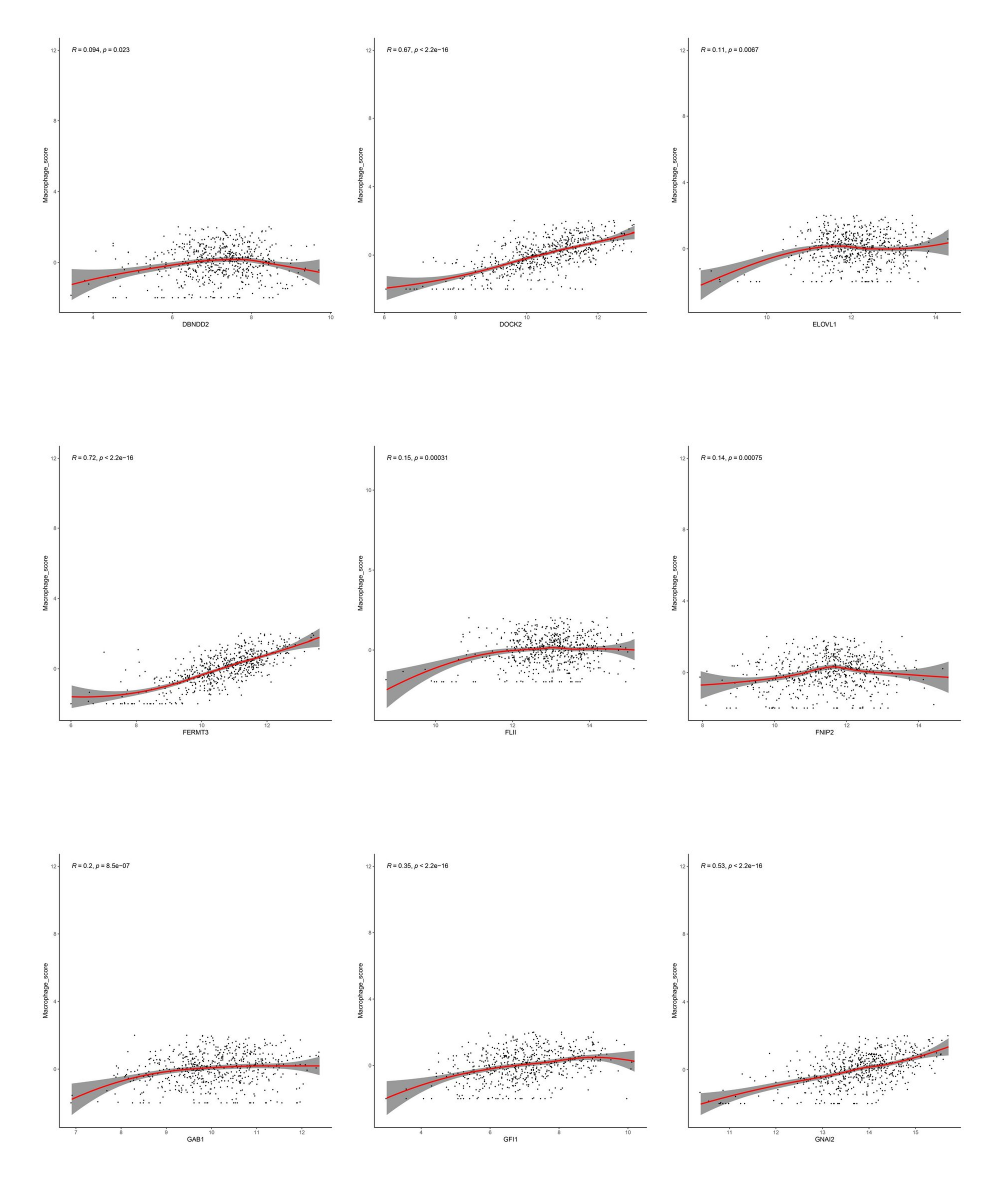

## Slide 4
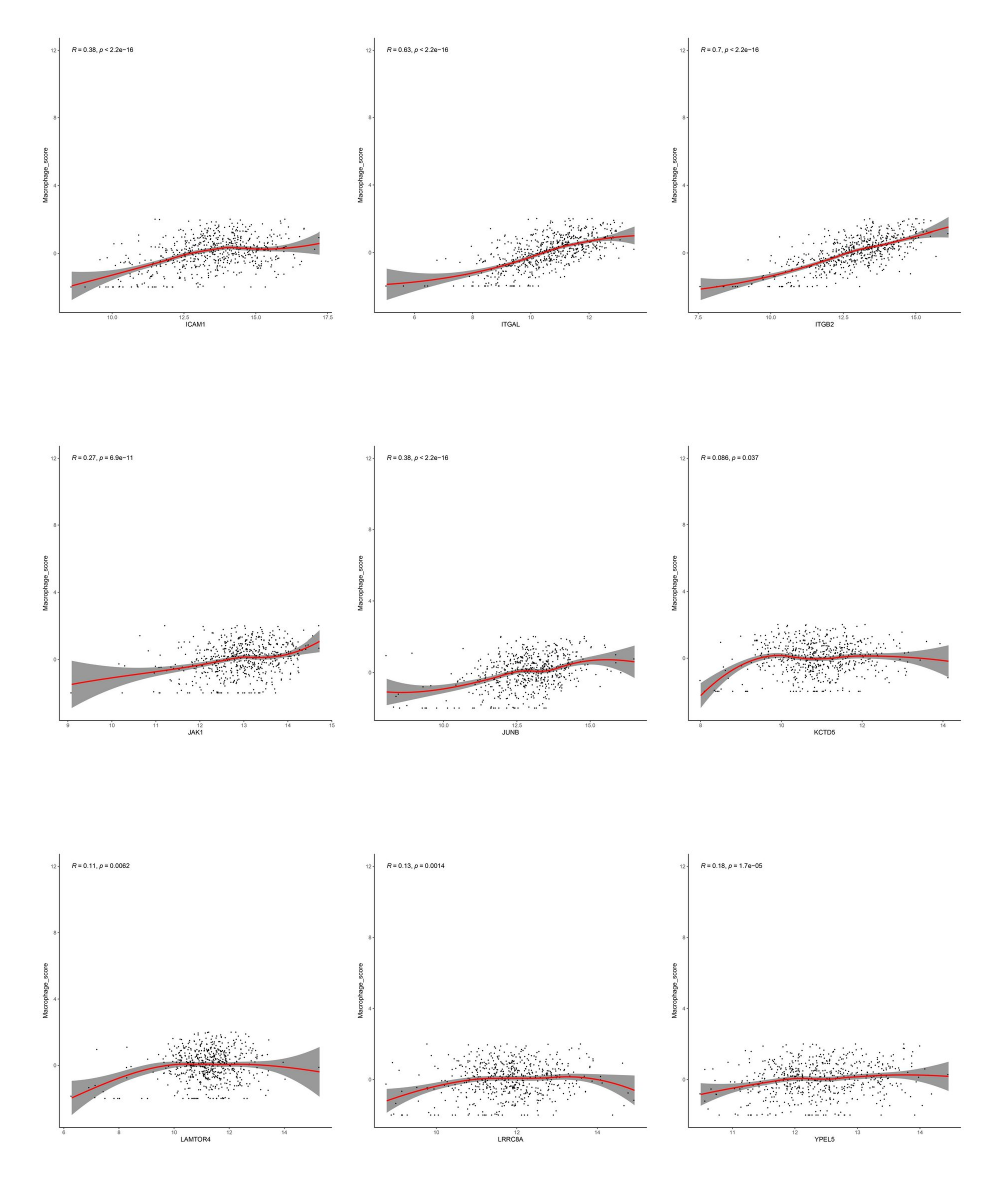

## Slide 5
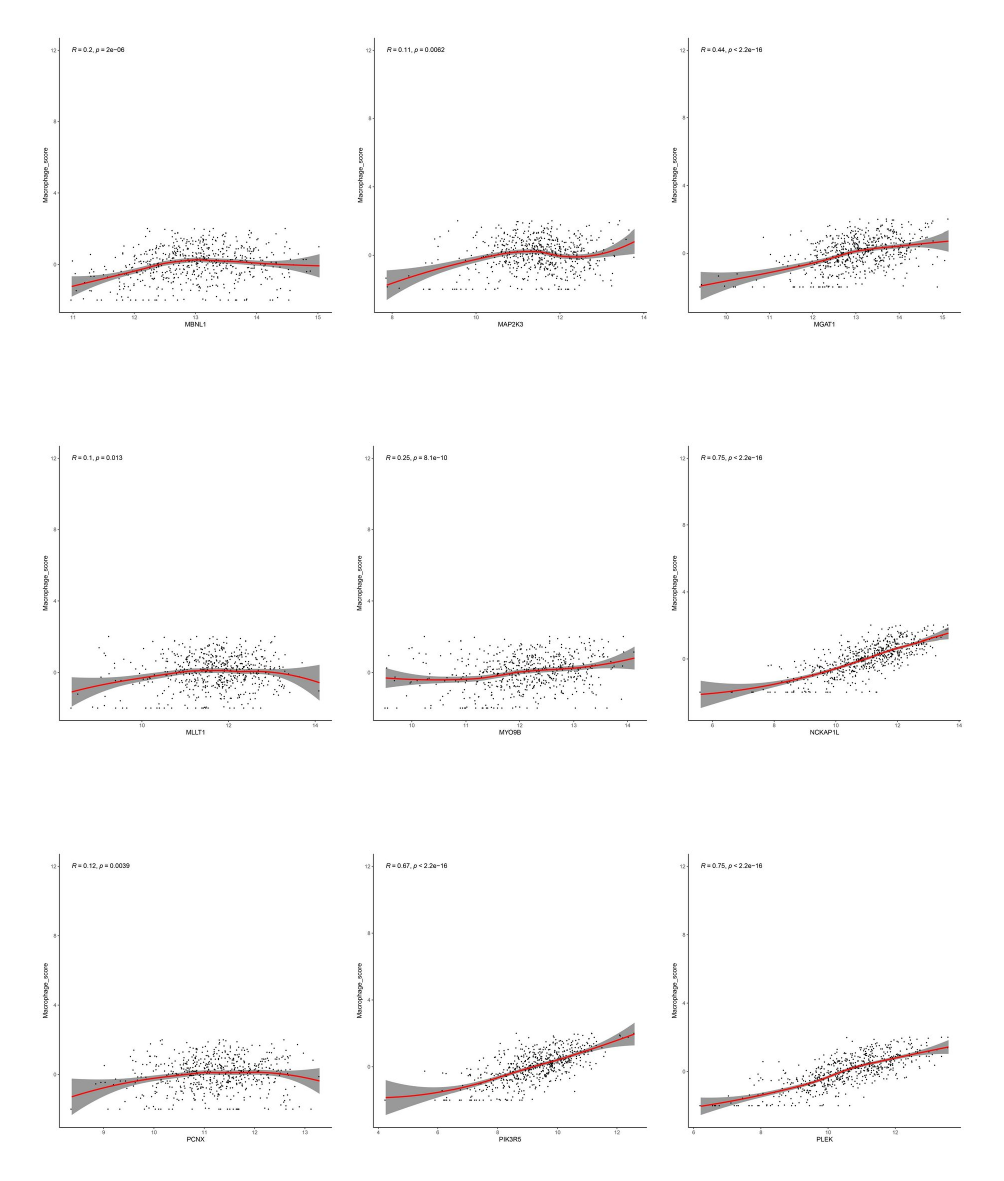

## Slide 6
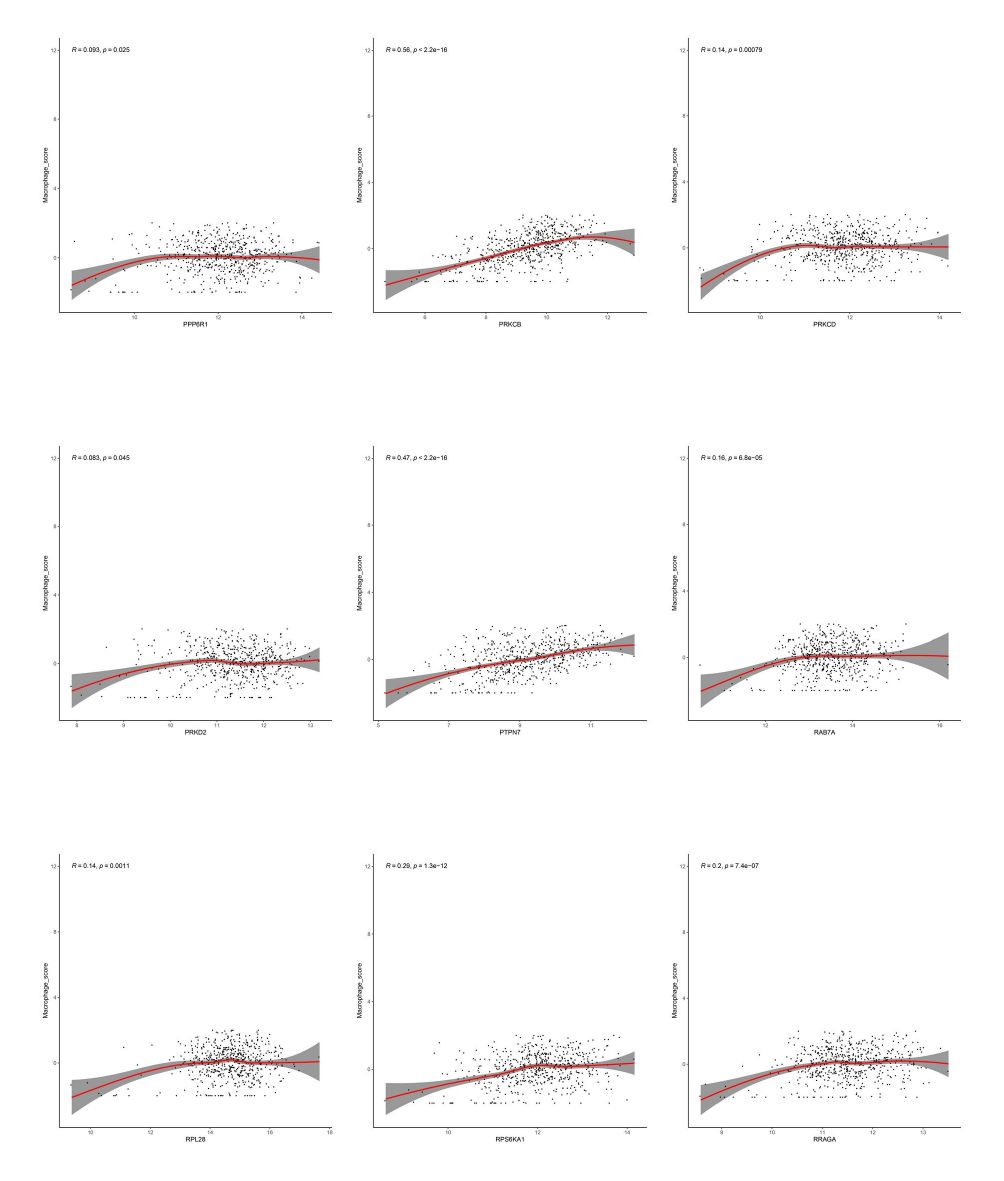

## Slide 7
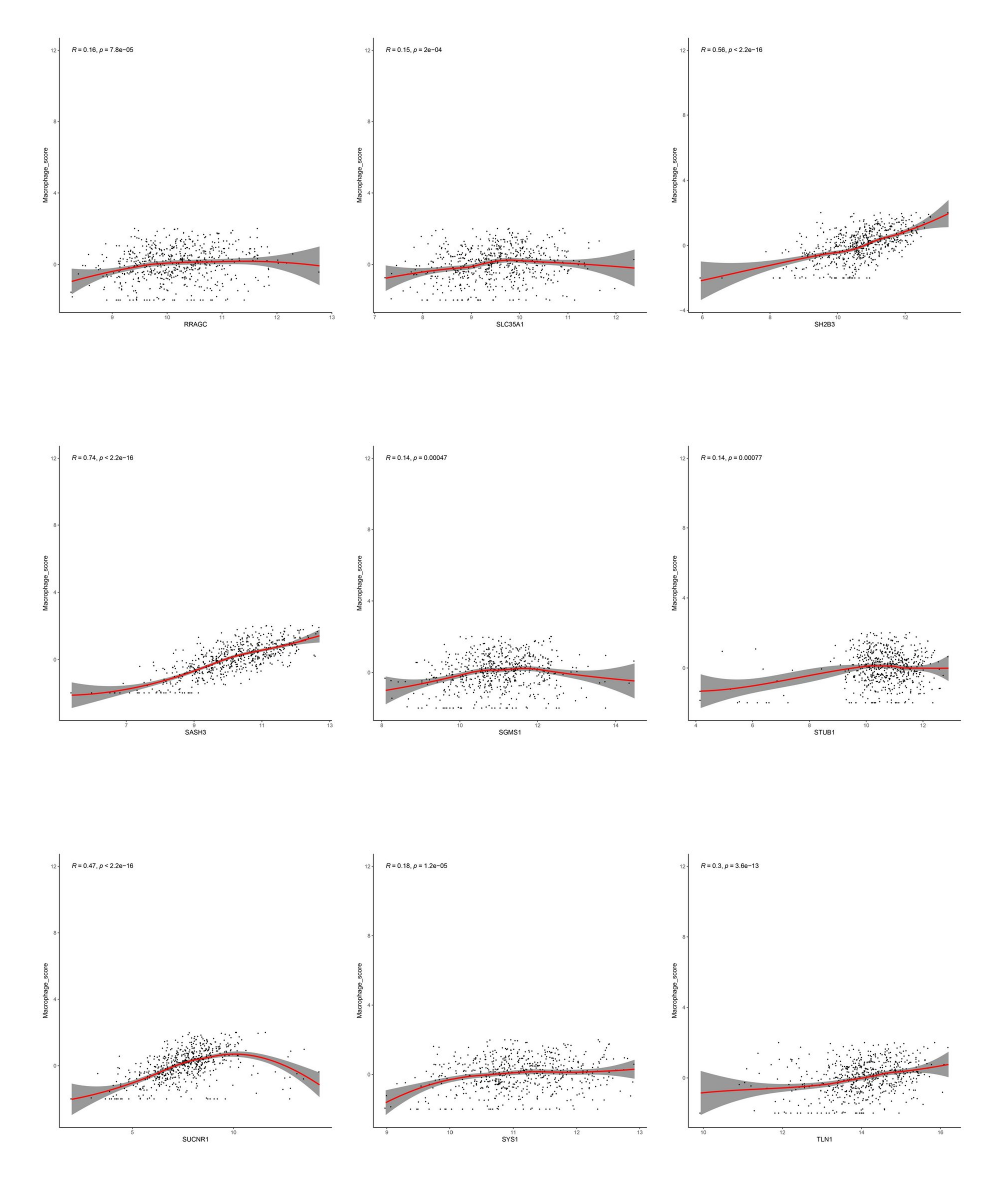

## Slide 8
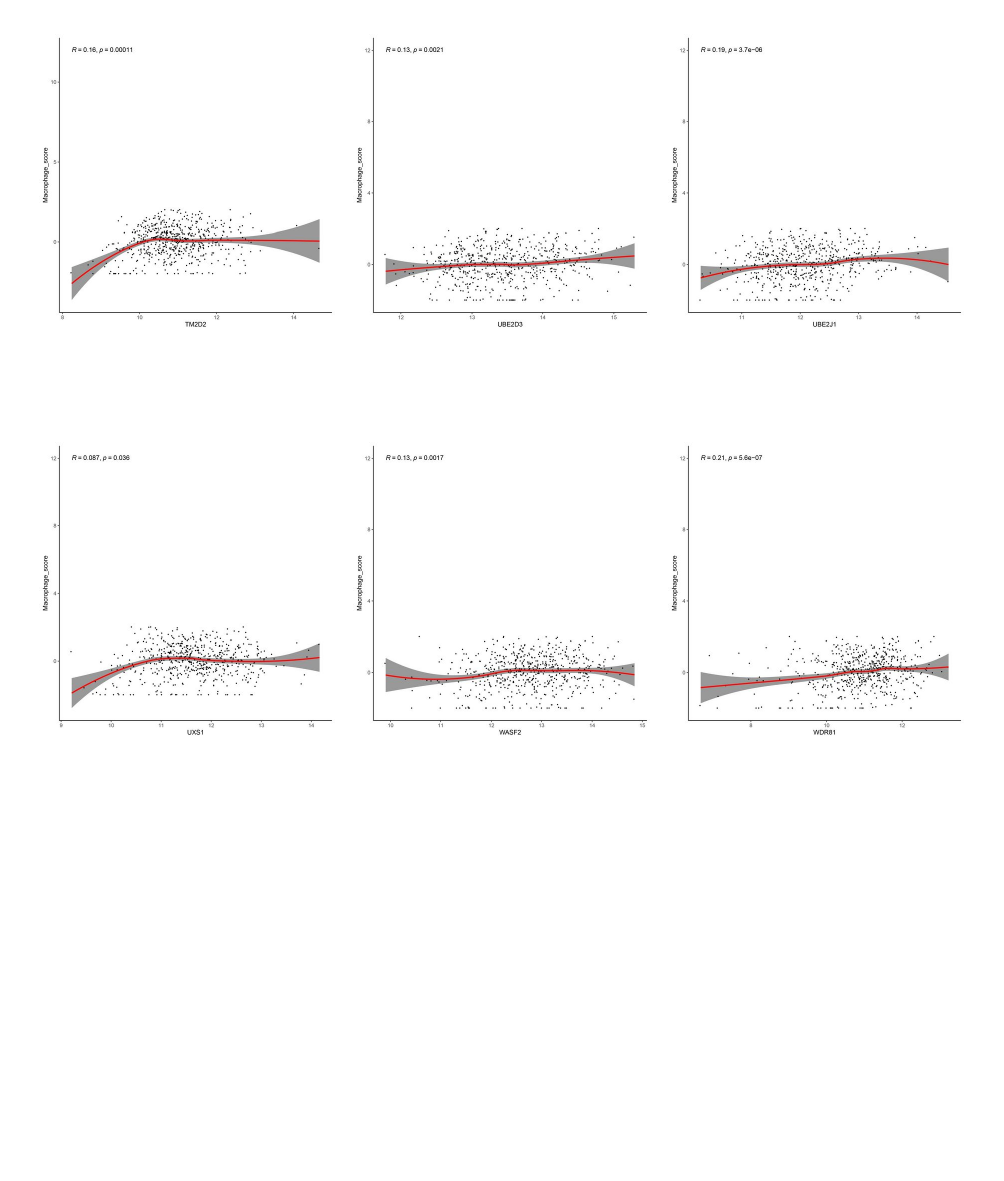

## Slide 9
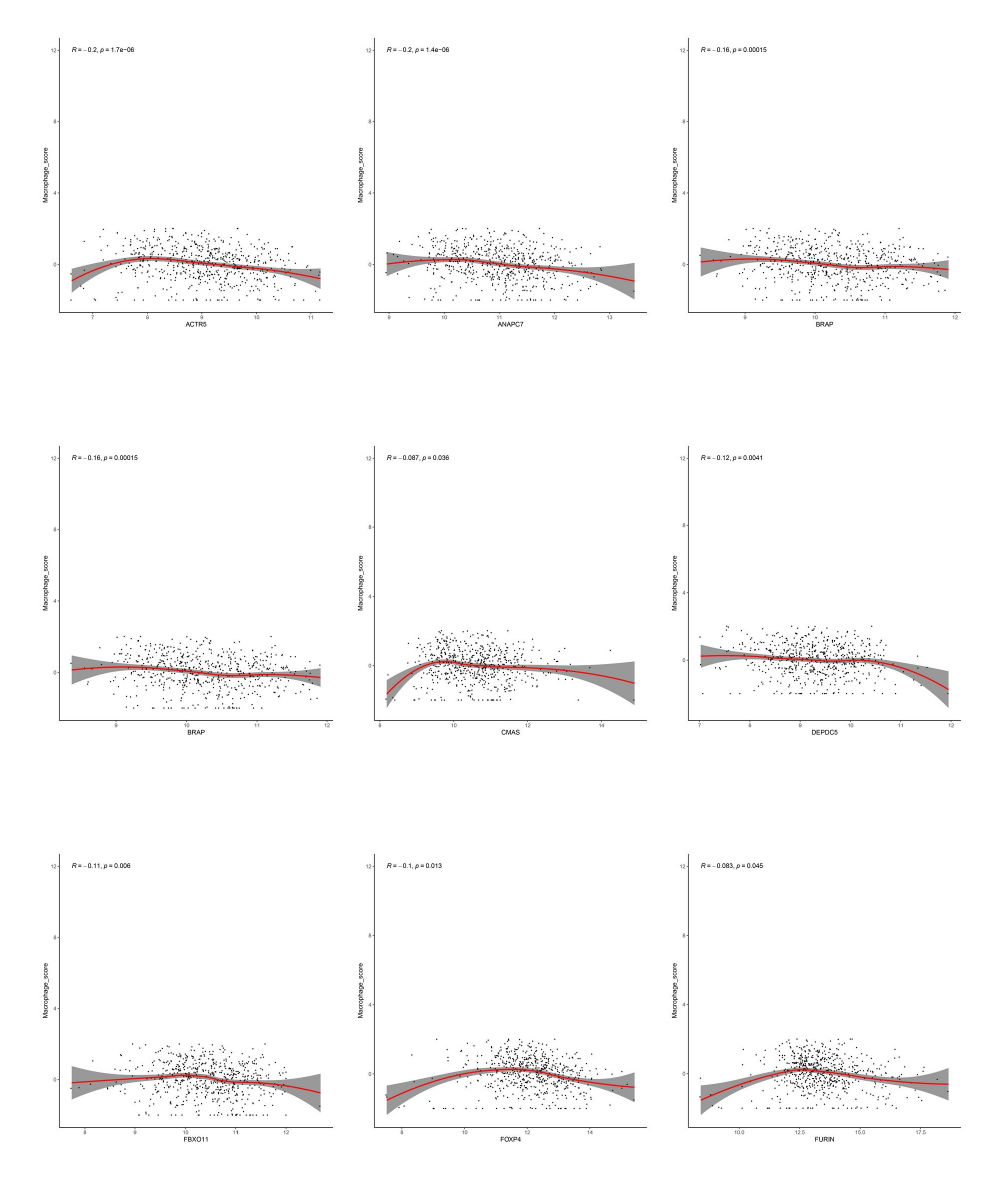

## Slide 10
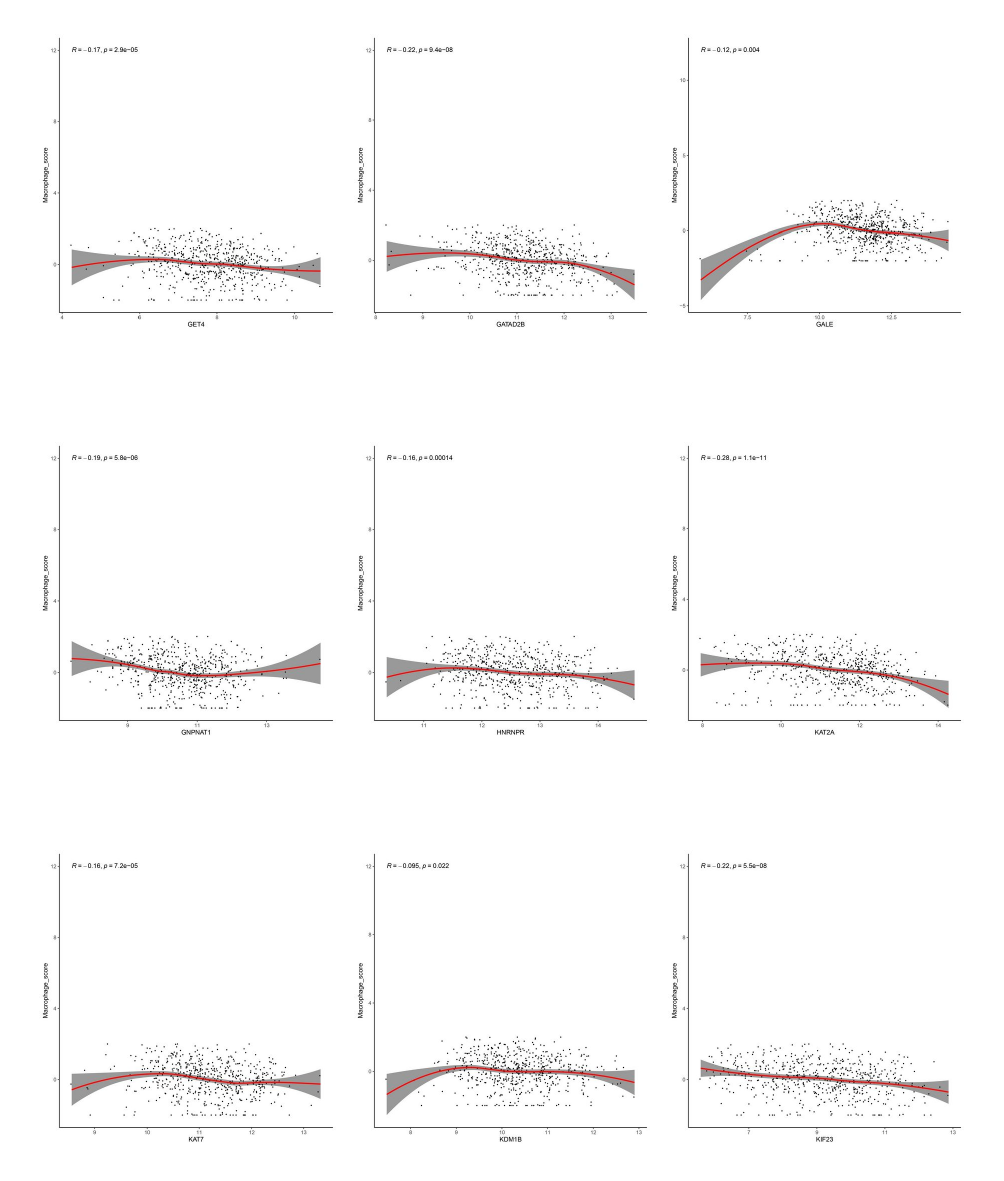

## Slide 11
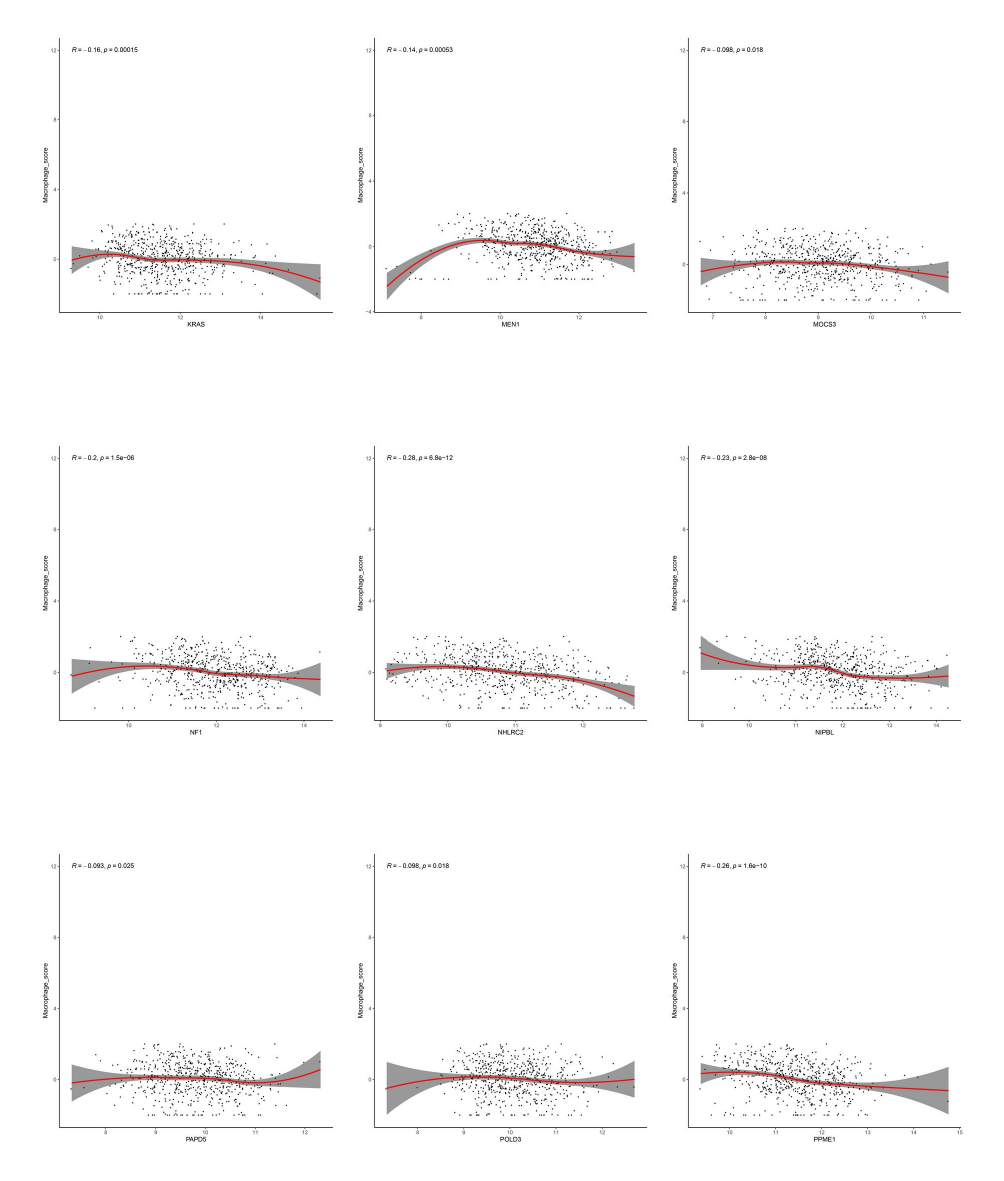

## Slide 12
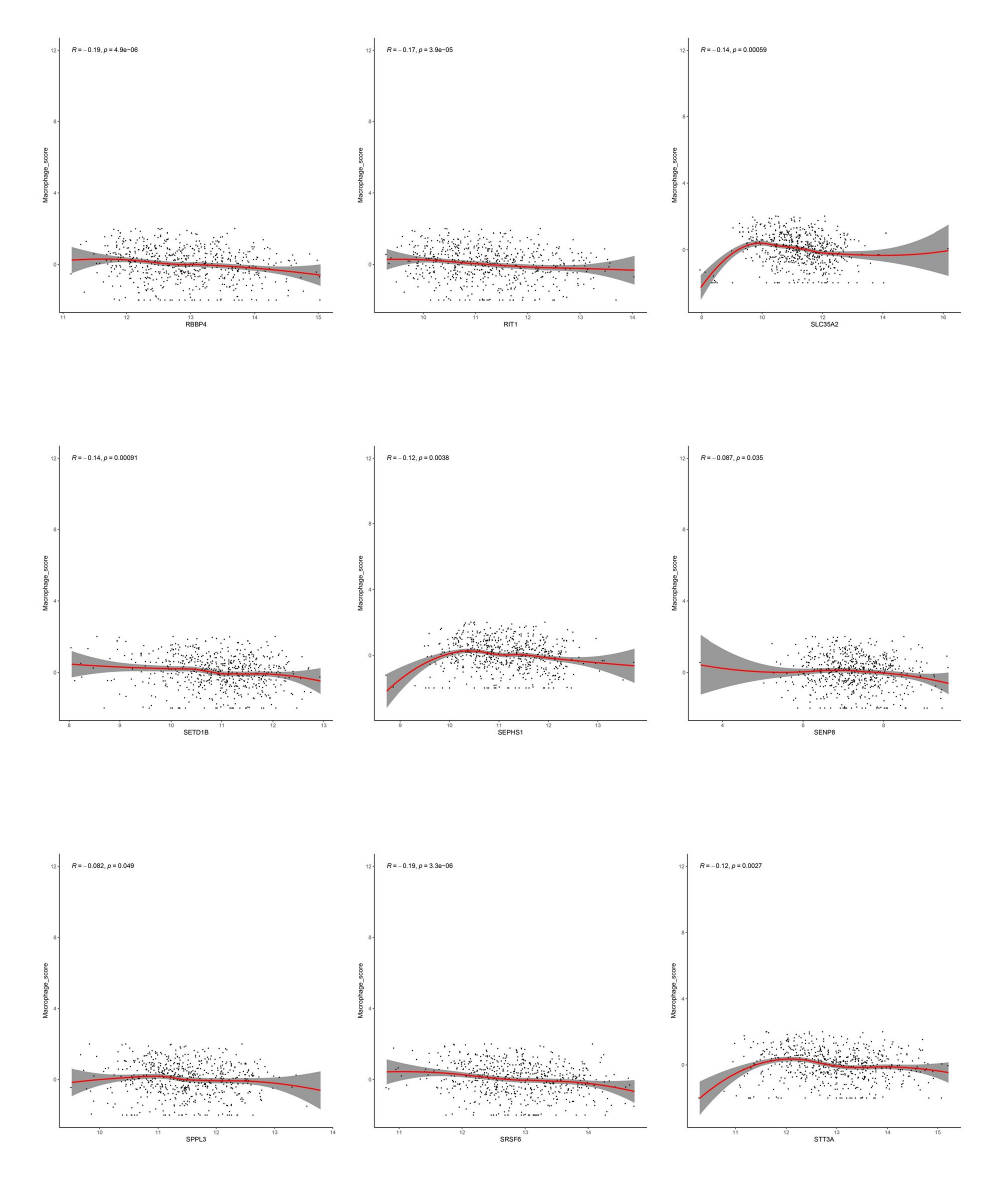

## Slide 13
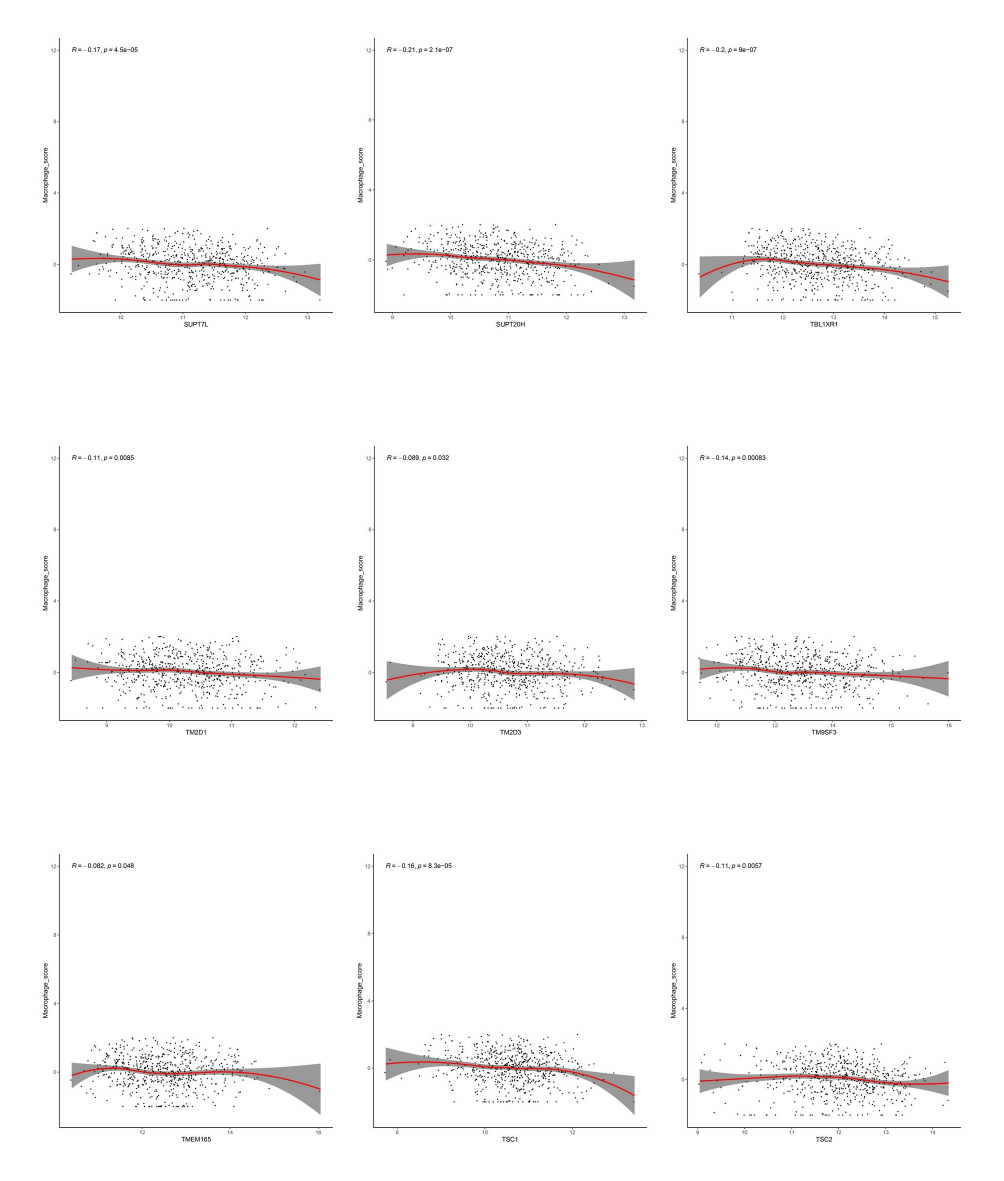

## Slide 14
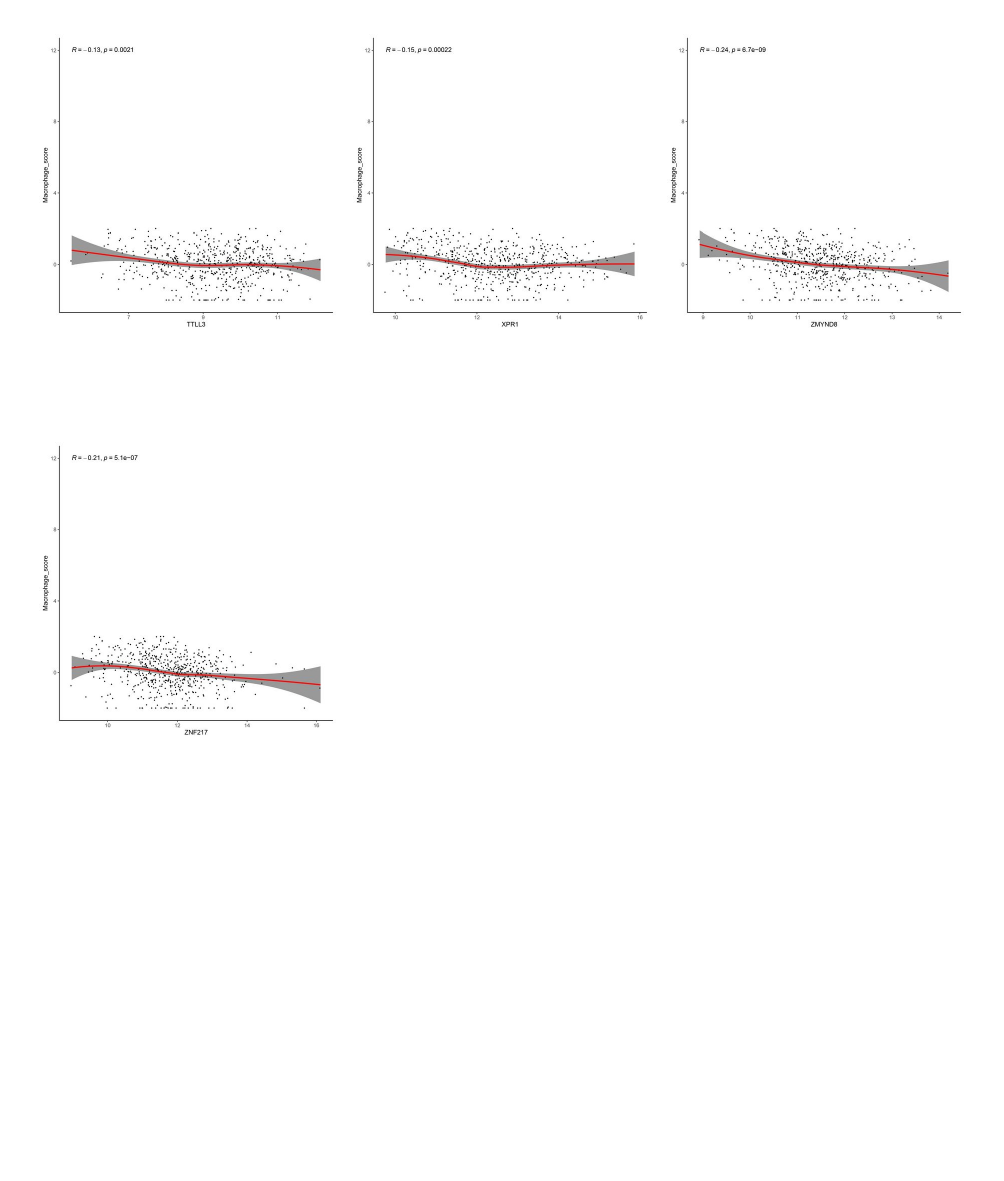

Supplement: Supplementary file 1 [file Presentation_1.pptx]

## Slide 1
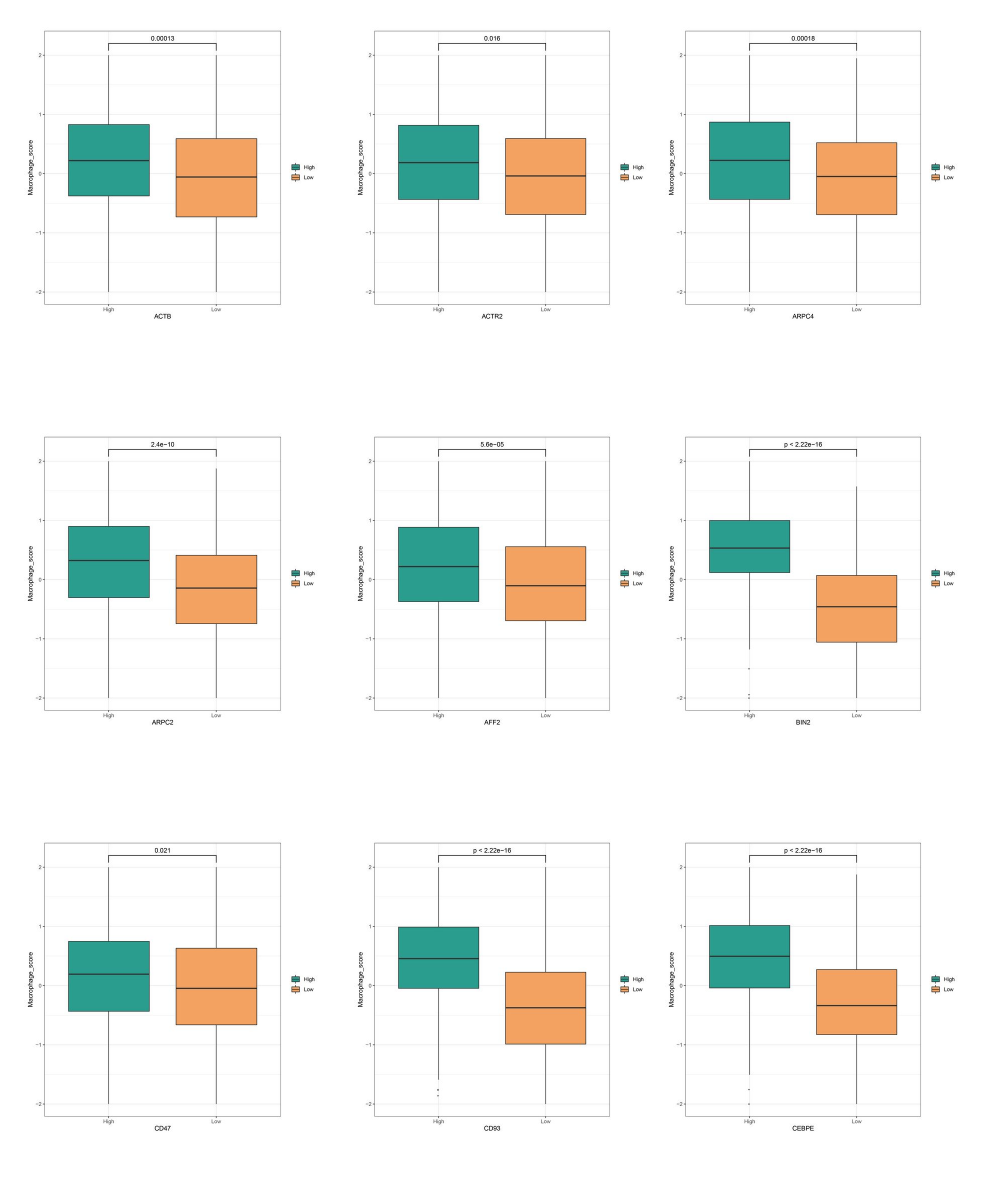

## Slide 2
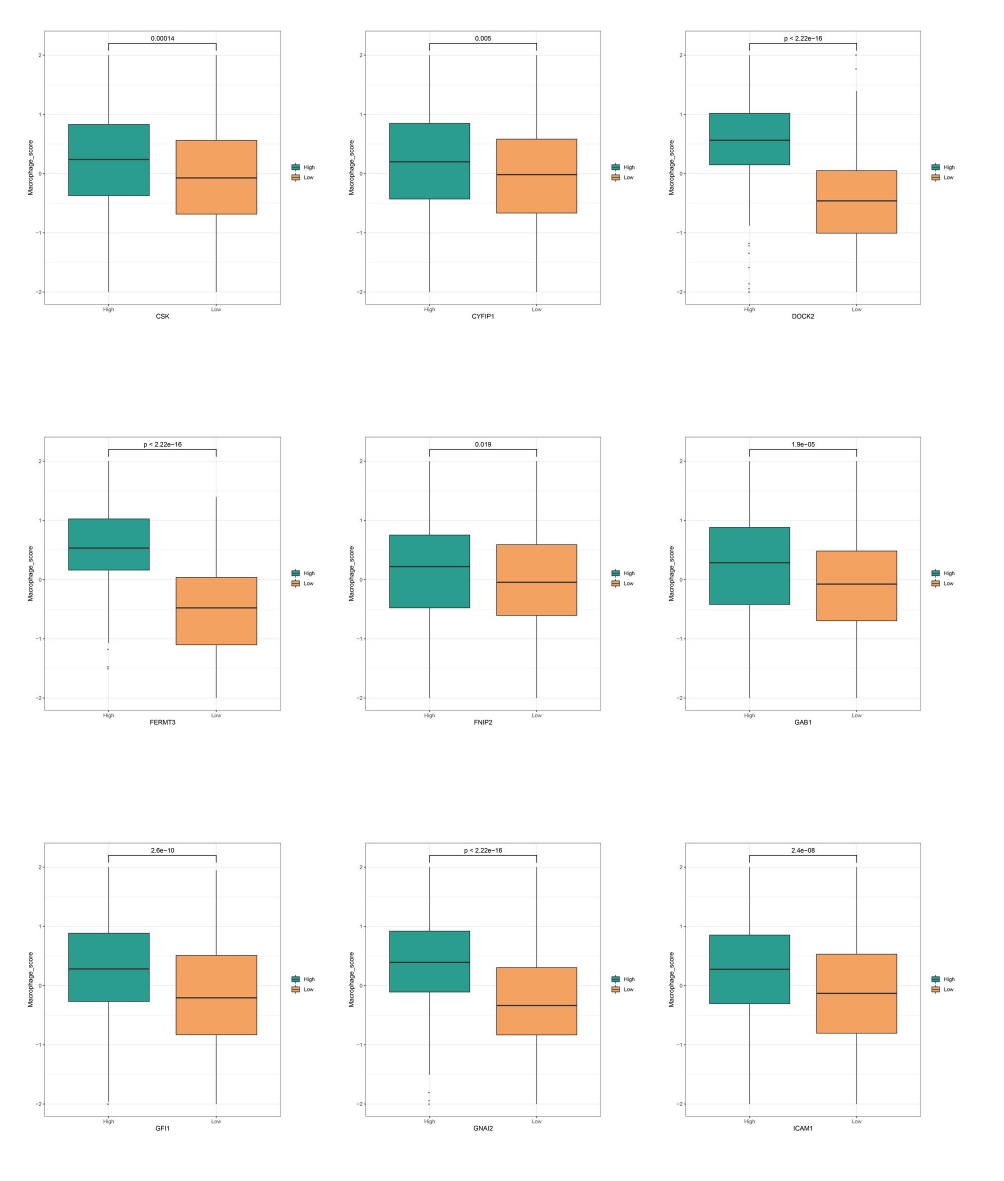

## Slide 3
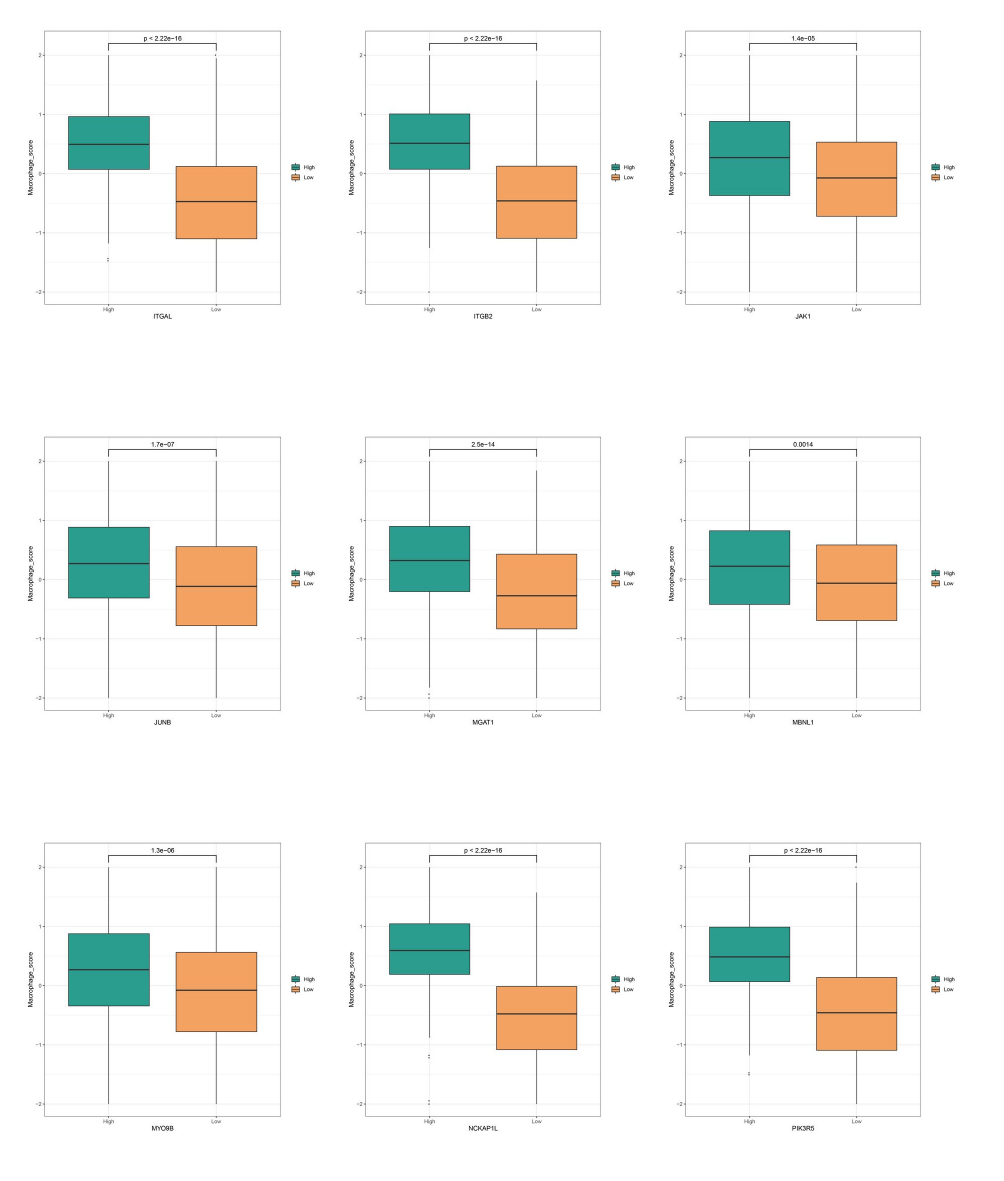

## Slide 4
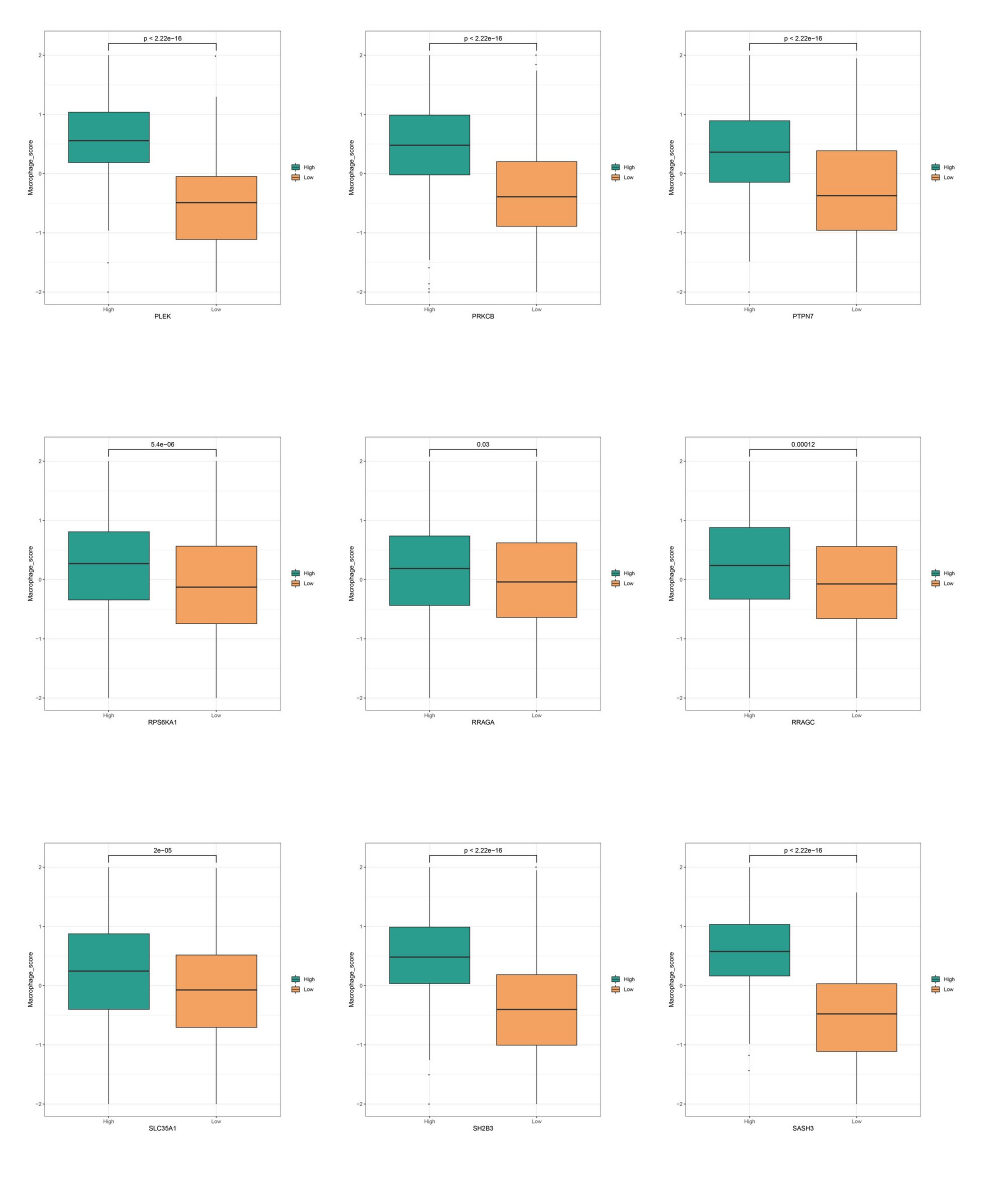

## Slide 5
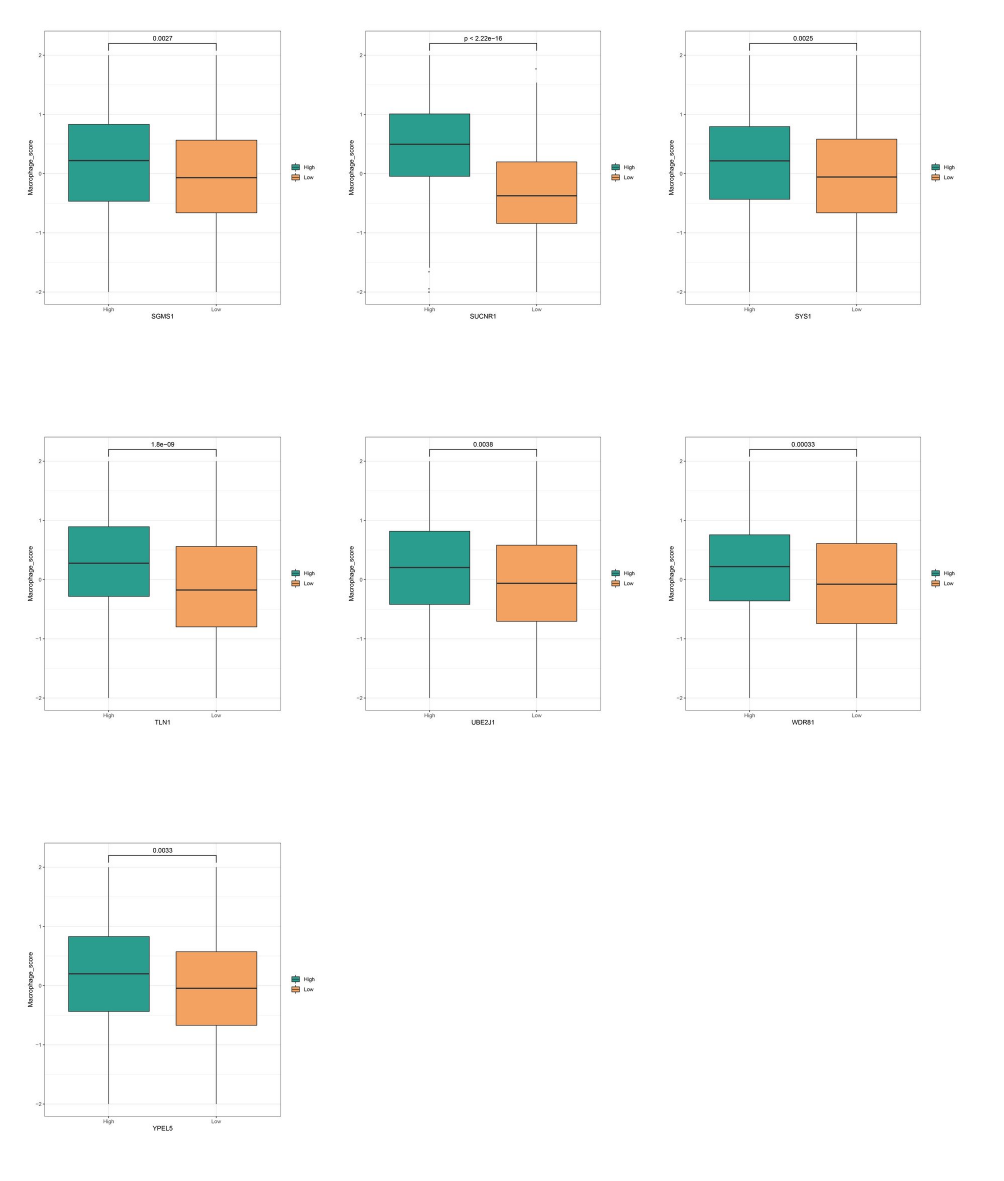

## Slide 6
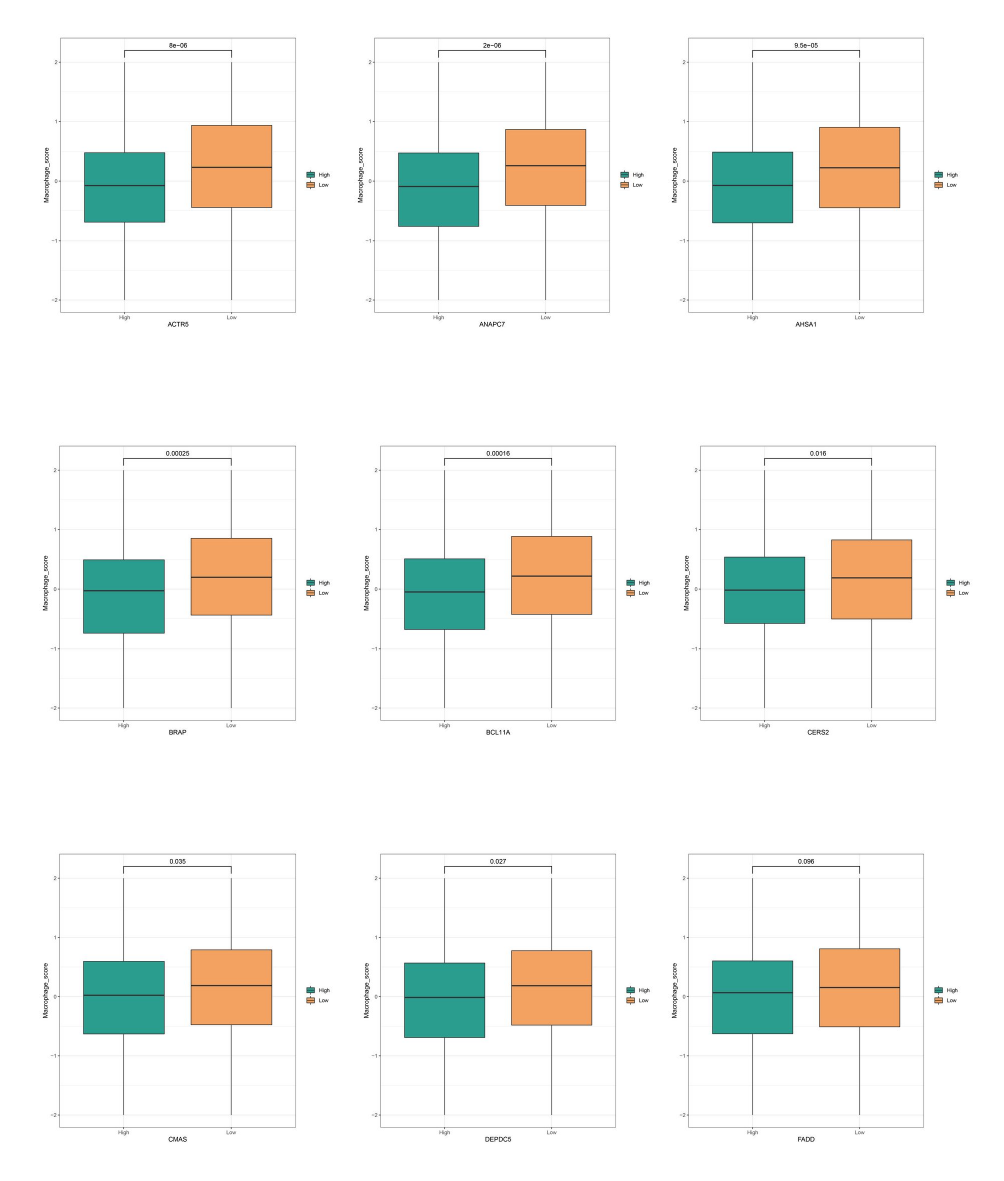

## Slide 7
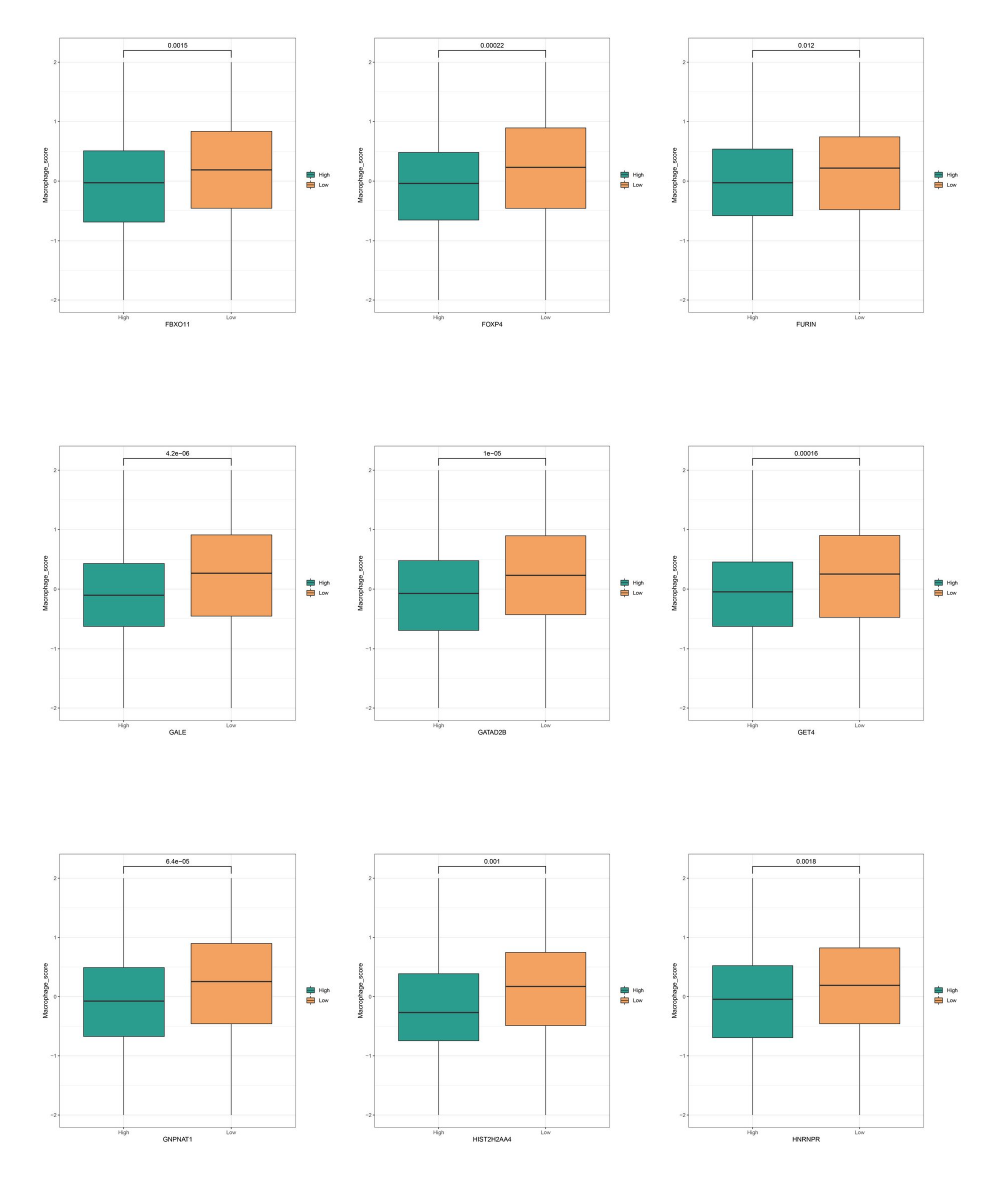

## Slide 8
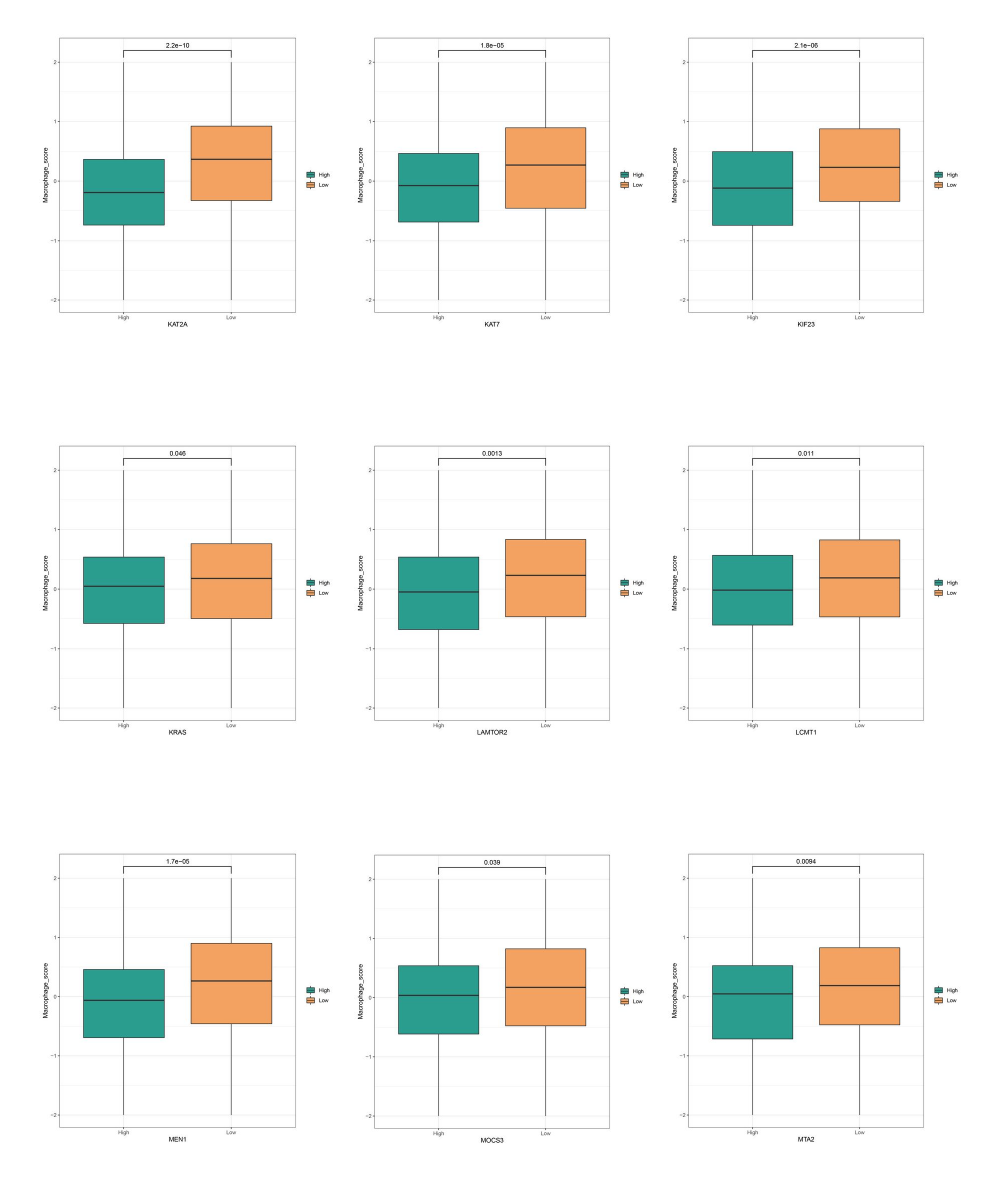

## Slide 9
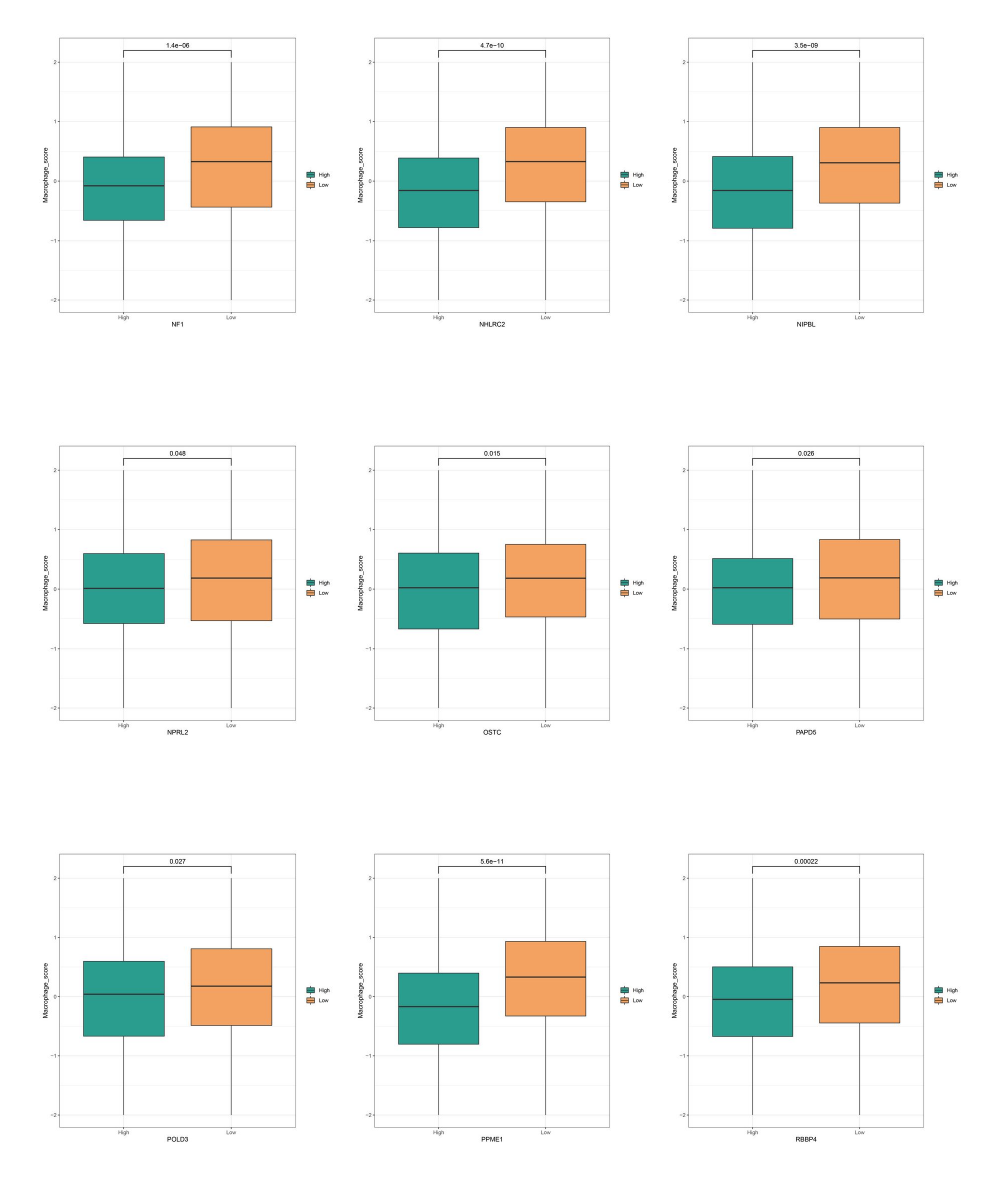

## Slide 10
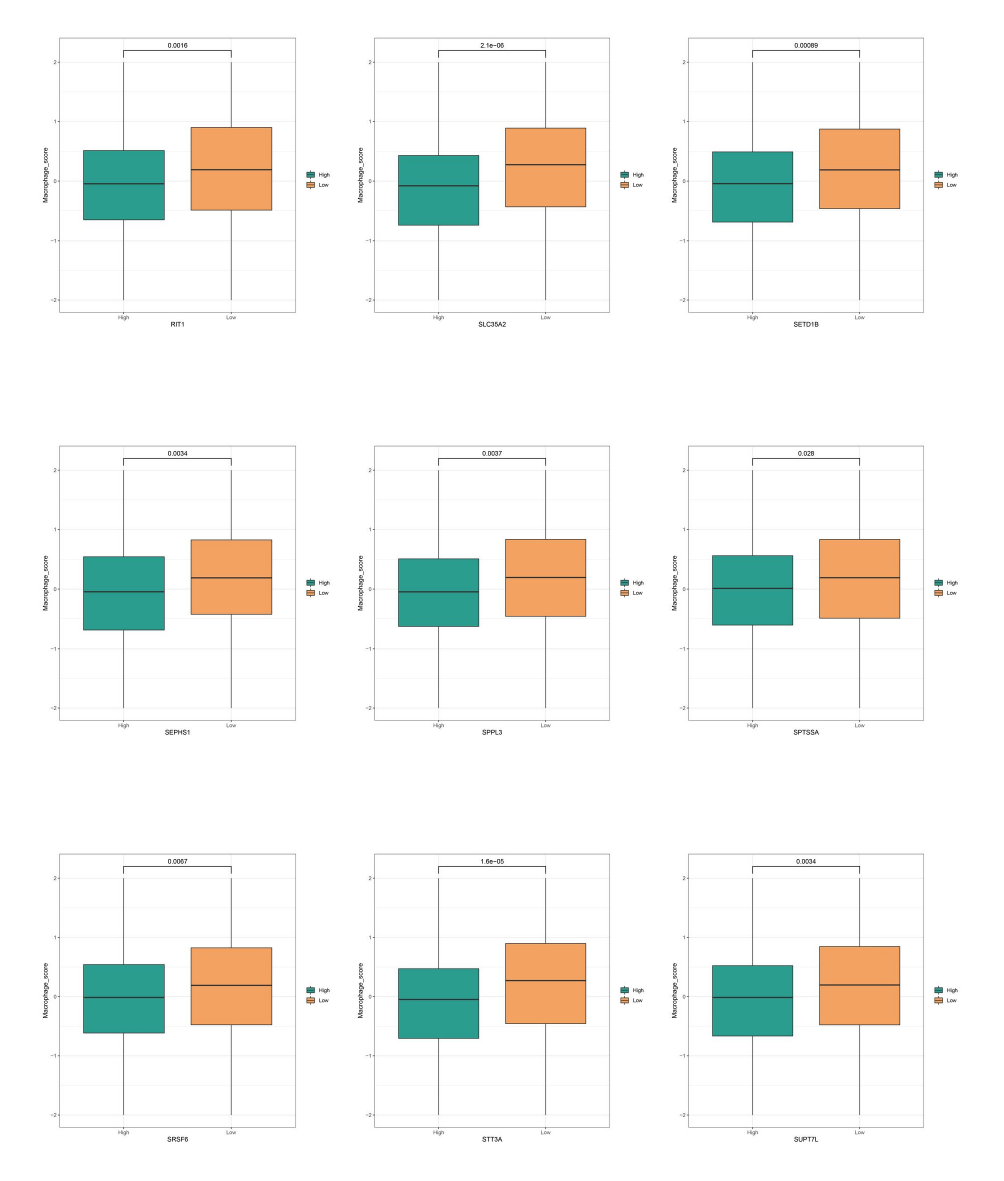

## Slide 11
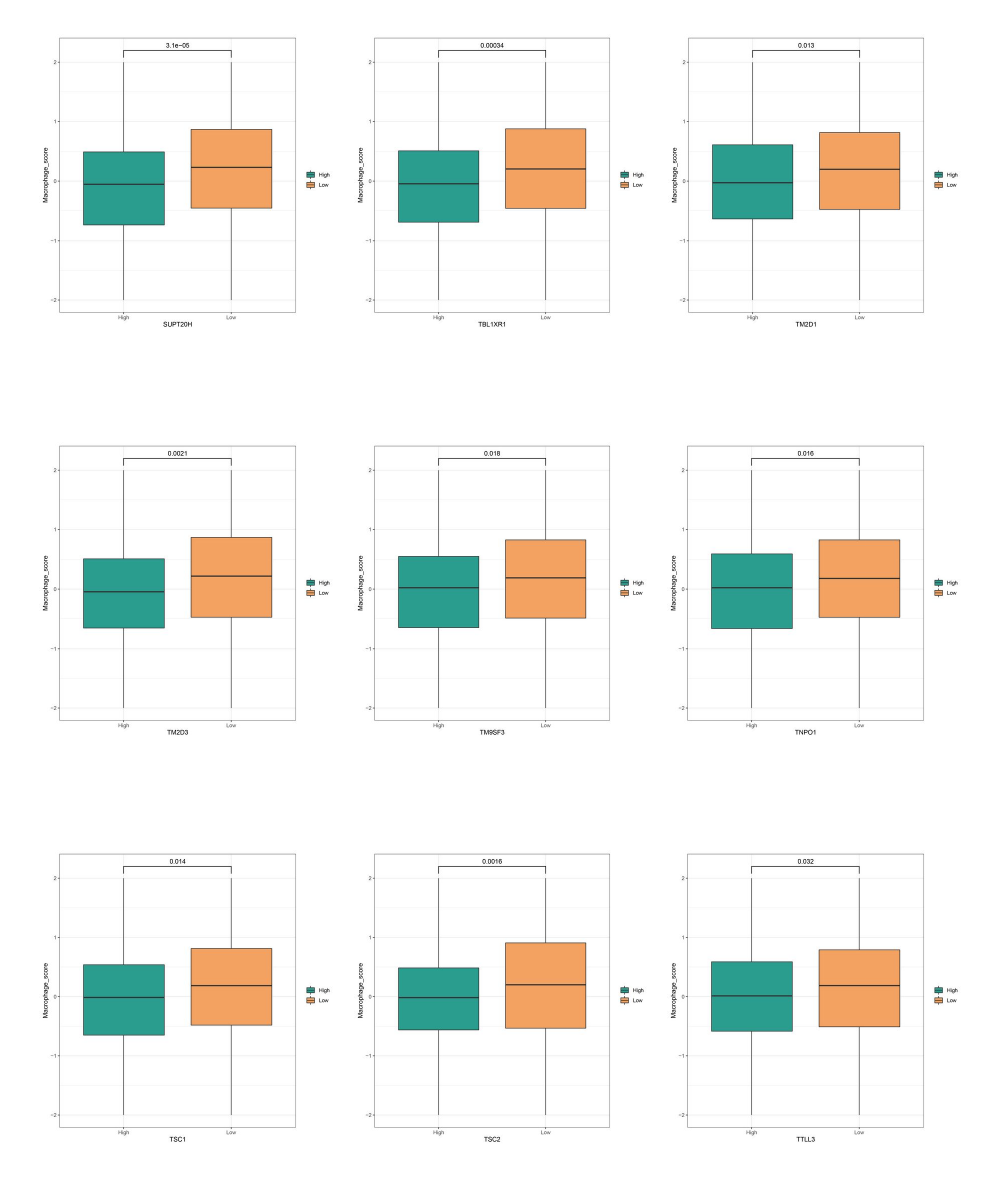

## Slide 12
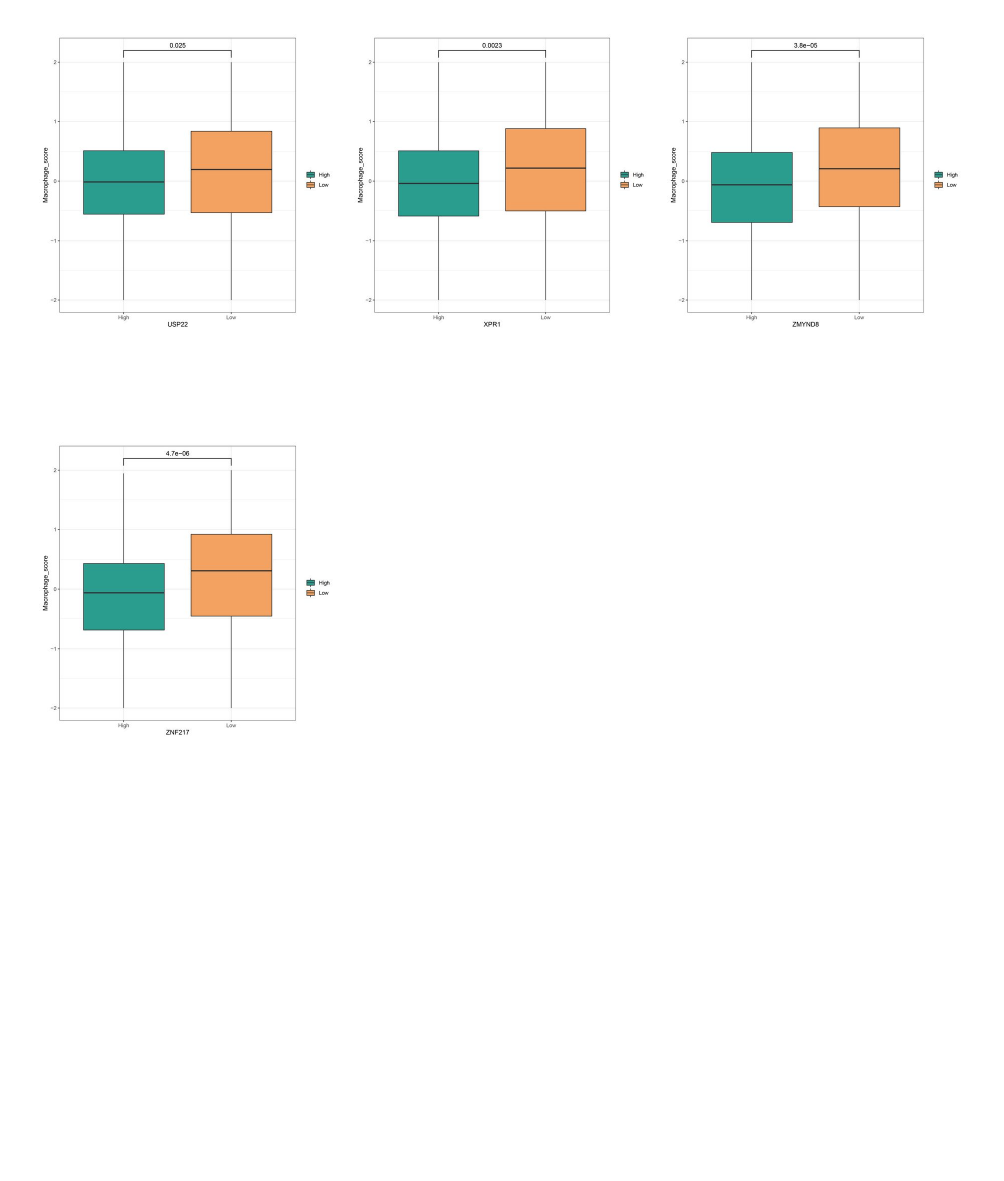

Supplement: Supplementary file 2 [file Presentation_2.pptx]

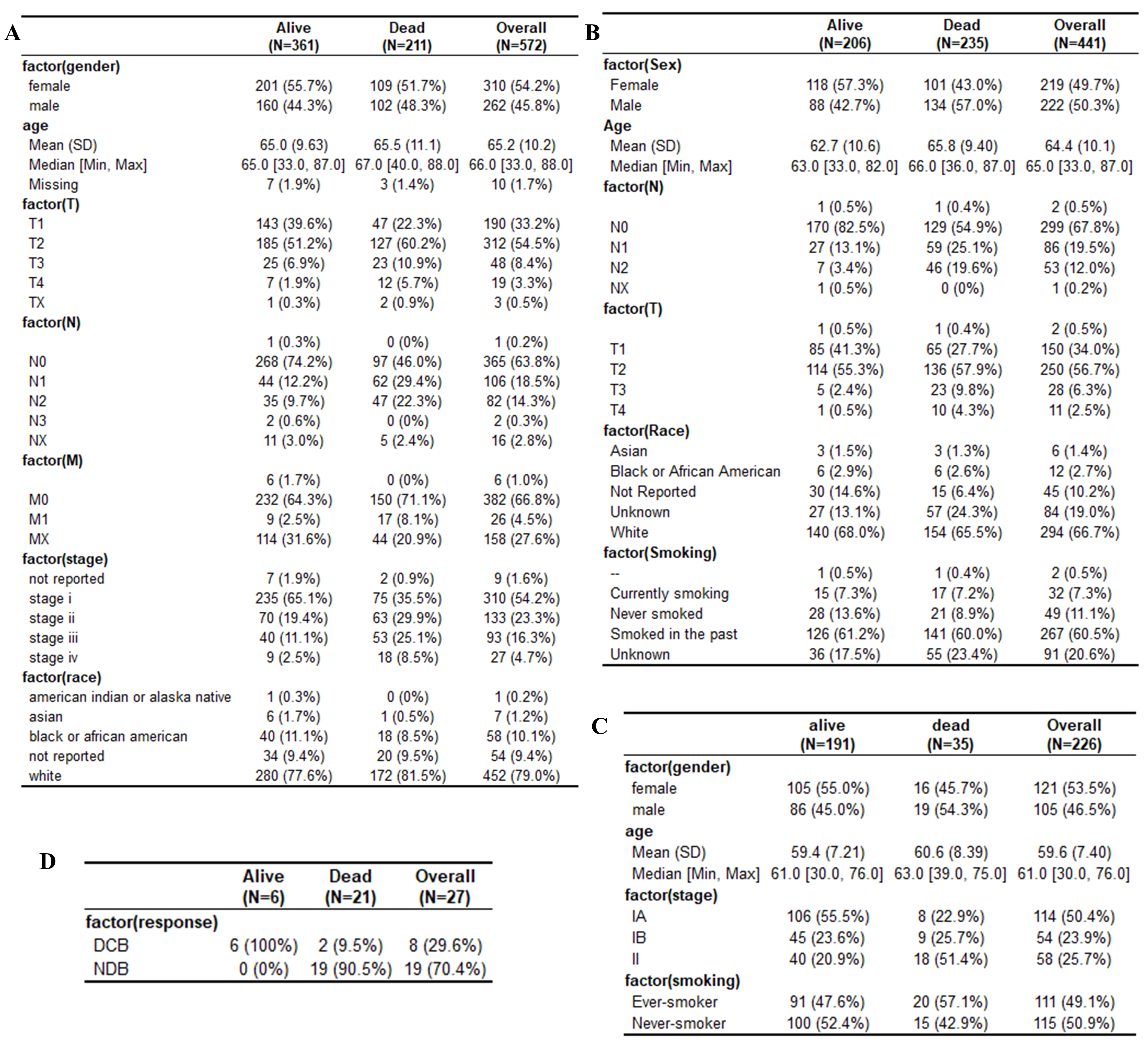

Supplement: Supplementary file 4 [file Image_1.tif]

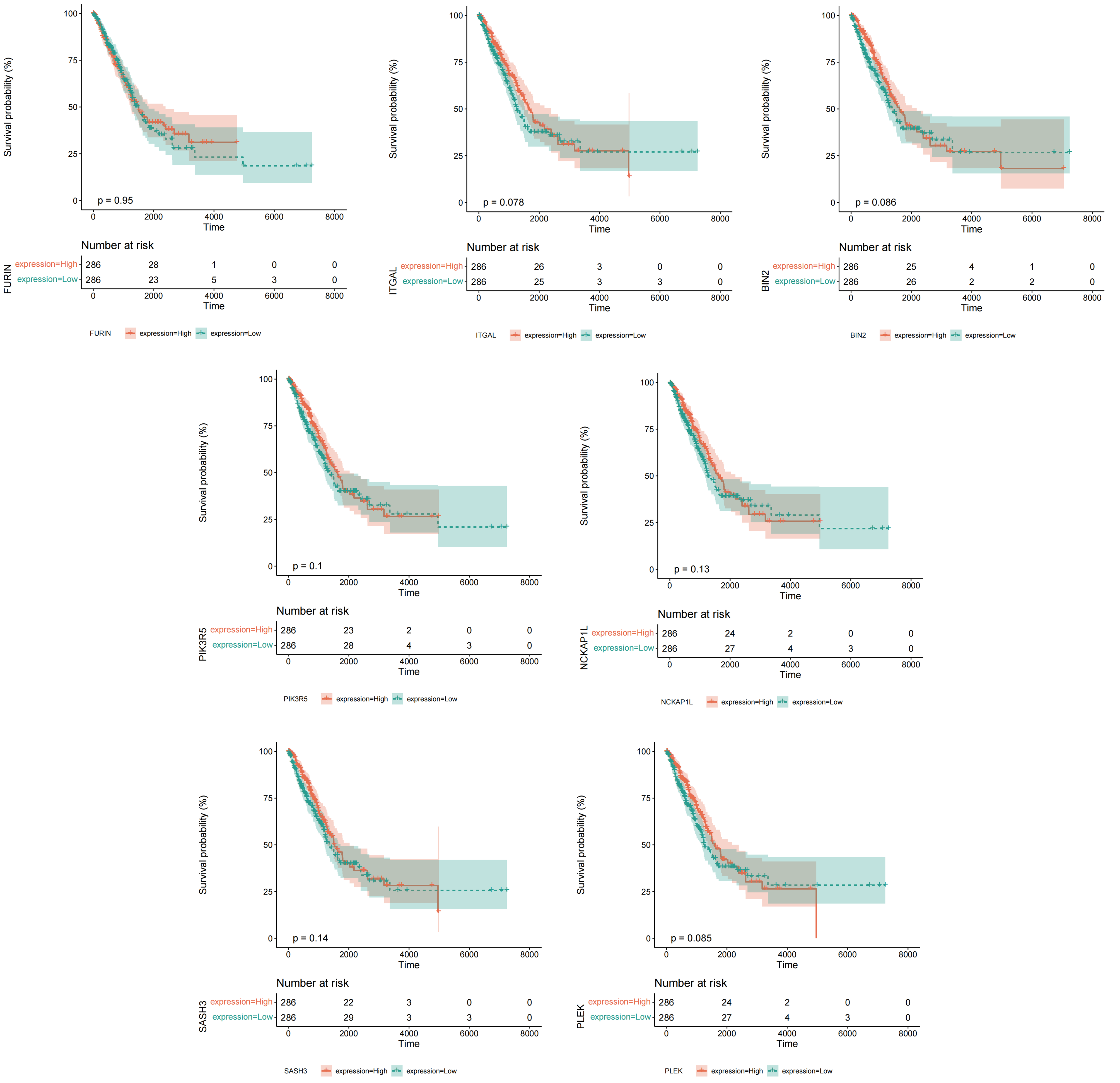

Supplement: Supplementary file 5 [file Image_2.tif]

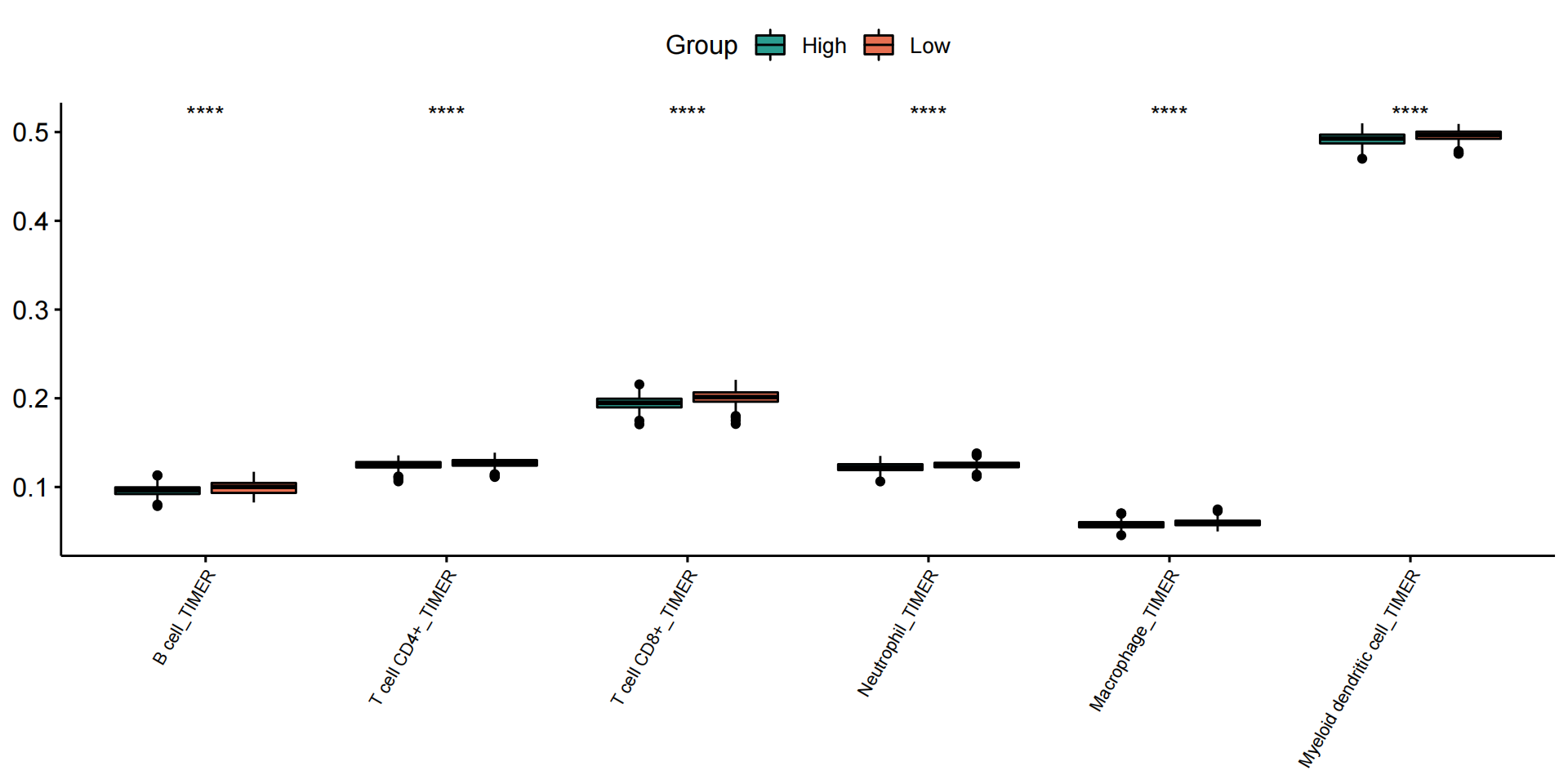

Supplement: Supplementary file 6 [file Image_3.tif]

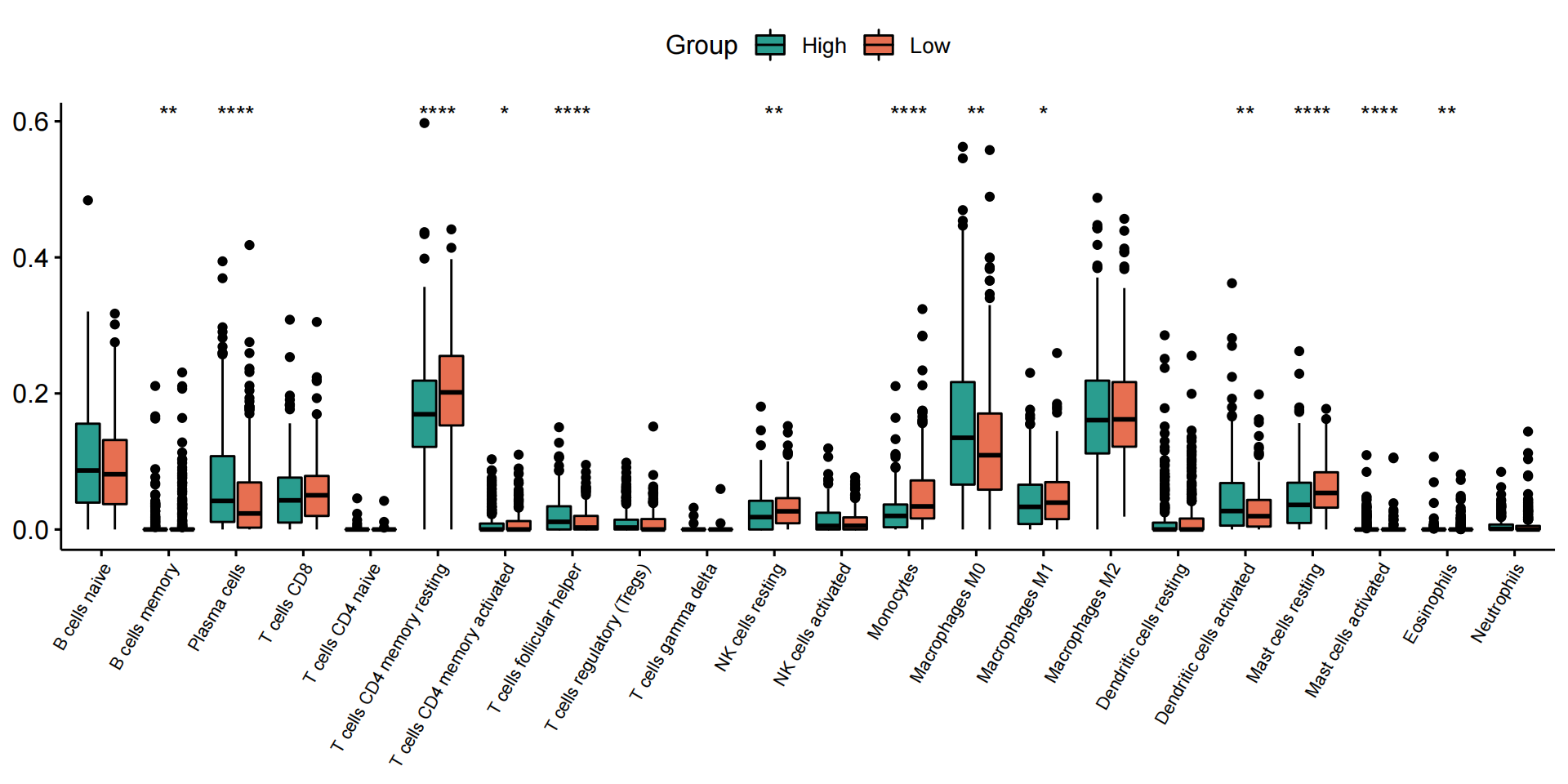

Supplement: Supplementary file 7 [file Image_4.tif]

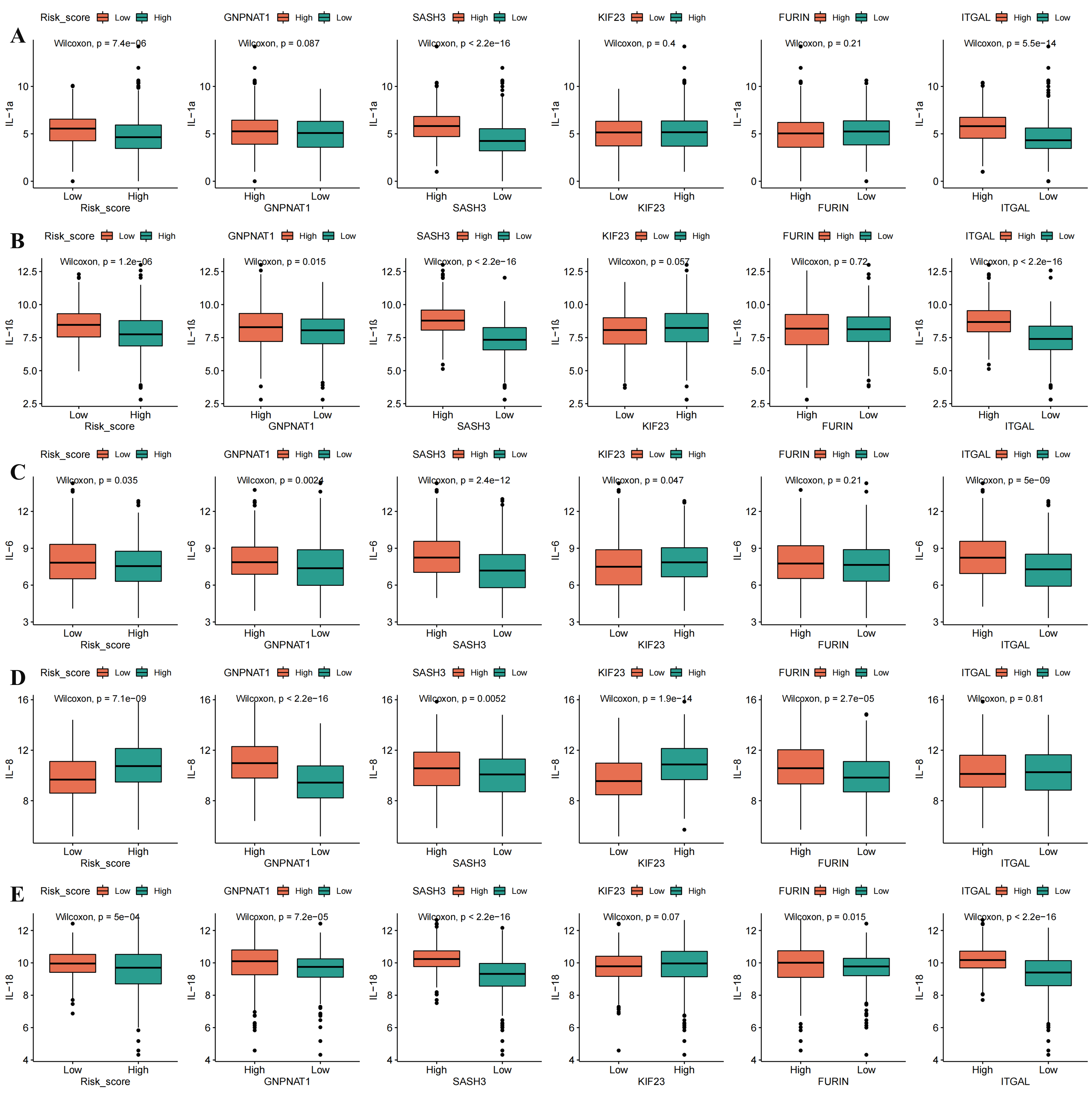

Supplement: Supplementary file 8 [file Image_5.tif]
