# Supplementary material for: Identification and validation of a novel phagocytosis regulators-related signature with potential prognostic and immunotherapeutic value in patients with lung adenocarcinoma
Source: Front Oncol. 2022 Nov 2;12:988332. doi: 10.3389/fonc.2022.988332 (PMC9666737; doi:10.3389/fonc.2022.988332)
Supplement: Supplementary file 3 [file DataSheet_1.docx]

**Supplementary materials**

**For**

**Identification and validation of novel phagocytosis regulators** **-related signature with potential prognostic and immunotherapeutic value in patients with lung adenocarcinoma**

*Jingyang Li^1^, Qinyun Du^1^, Jiayi Sun^2^, Li Xiang^2*^*, *Shaohui Wang^3*^*

*^1^State Key Laboratory of Southwestern Chinese Medicine Resources, School of Pharmacy, Chengdu University of Traditional Chinese Medicine, Chengdu, China,* *^2^State Key Laboratory of Southwestern Chinese Medicine Resources, Innovative Institute of Chinese Medicine and Pharmacy, Chengdu University of Traditional Chinese Medicine, Chengdu, China**, ^3^State Key Laboratory of Southwestern Chinese Medicine Resources, School of Ethnic Medicine,* *Chengdu University of Traditional Chinese Medicine, Chengdu, China*

**Corresponding authors:** Shaohui Wang (winter9091@163.com); Li Xiang (xianglydr@cdutcm.edu.cn); Xianli Meng (xlm999@cdutcm.edu.cn)

**Supplementary Figure 1.** The clinical characteristics of LUAD patients in the TCGA **(A)**, GSE68465 **(B)**, GSE31210 **(C)**, and GSE135222 **(D)** dataset.

**Supplementary Figure 2.** Correlation analysis of phagocytosis regulators expression level and macrophage enrichment score in LUAD samples.

**Supplementary Figure 3.** Grouping comparison of phagocytosis regulators and macrophage enrichment scores.

**Supplementary Figure 4.** Kaplan-meier curves of phagocytosis regulators (Including ITGAL, SASH3, BIN2, FURIN, NCKAP1L, PLEK and PIK3R5) .

**Supplementary Figure 5.** The expression differences of different immune cell infiltrates between the high and low risk groups were analyzed by TIMER algorithm.

**Supplementary Figure 6.** The expression differences of different immune cell infiltrates between the high and low risk groups were analyzed by Cibersort algorithm.

**Supplementary Figure 7.** Correlation analysis between risk score, five prognostic phagocytosis regulators signatures and proinflammatory factor group. **(A)** IL-1α. **(B)** IL-1β. **(C)** IL-6. **(D)** IL-8 and **(E)** IL-18.

**Supplementary Table S1. The primer sequences used in this study.**

| **Primer** | **Sequence (5’-3’)** |
| --- | --- |
| GNPNAT1-F | ACTCCTATGTTTGACCCAAGTCT |
| GNPNAT1-R | TCTGTTAGCTGACCCAATACCT |
| SASH3-F | AAGGTGGGCTCTTTCAAATTCA |
| SASH3-R | CATGCAGGGTCTTAGGCTTGG |
| KIF23-F | TGTGGCTAATCCCTTGGTCAA |
| KIF23-R | AGAACCAGTCATTGTGTGAGTTT |
| FURIN-F | TCGGGGACTATTACCACTTCTG |
| FURIN-R | CCAGCCACTGTACTTGAGGC |
| ITGAL-F | TGCTTATCATCATCACGGATGG |
| ITGAL-R | CTCTCCTTGGTCTGAAAATGCT |
| GADPH-sense | CCTGGTATGACAACGAATTTG |
| GADPH-antisense | CAGTGAGGGTCTCTCTCTTCC |

**Supplementary Table S2.**

| **NO.** | **gene name** | **NO.** | **gene name** | **NO.** | **gene name** | **NO.** | **gene name** | **NO.** | **gene name** | **NO.** | **gene name** | **NO.** | **gene name** |
| --- | --- | --- | --- | --- | --- | --- | --- | --- | --- | --- | --- | --- | --- |
| **1** | NHLRC2 | **31** | MTA2 | **61** | SLC35A1 | **91** | ZMYND8 | **121** | SETD1B | **151** | SUPT7L | **181** | CSNK1A1 |
| **2** | TM2D2 | **32** | CCNC | **62** | TSC2 | **92** | ELOVL1 | **122** | KDM1B | **152** | ARIH2 | **182** | CD47 |
| **3** | TLN1 | **33** | CHIC2 | **63** | GNE | **93** | SEPHS1 | **123** | LRRC8A | **153** | SENP8 | **183** | APMAP |
| **4** | FERMT3 | **34** | PCNX | **64** | UXS1 | **94** | RAC1 | **124** | BRPF1 | **154** | UBE2F |  |  |
| **5** | LCMT1 | **35** | TM2D1 | **65** | MAPK1 | **95** | MBNL1 | **125** | C11ORF83 | **155** | RRAGC |  |  |
| **6** | ITGAL | **36** | C11ORF73 | **66** | TSC1 | **96** | MGAT1 | **126** | RAF1 | **156** | CSK |  |  |
| **7** | ICAM1 | **37** | CD93 | **67** | MTR | **97** | SPTSSA | **127** | USP22 | **157** | MAP2K3 |  |  |
| **8** | ITGB2 | **38** | AHSA1 | **68** | DOCK2 | **98** | MLLT1 | **128** | FOXP4 | **158** | ACTB |  |  |
| **9** | SYS1 | **39** | FURIN | **69** | STT3A | **99** | SUCNR1 | **129** | CR769776.1 | **159** | BRAP |  |  |
| **10** | DBNDD2 | **40** | MYO9B | **70** | TMEM165 | **100** | CERS2 | **130** | AGFG1 | **160** | ACTR5 |  |  |
| **11** | TLE3 | **41** | BCL11A | **71** | RASA2 | **101** | RIT1 | **131** | PAPD5 | **161** | RAB7A |  |  |
| **12** | TM2D3 | **42** | KCTD5 | **72** | GALE | **102** | TTLL3 | **132** | BIN2 | **162** | OSTC |  |  |
| **13** | ABI1 | **43** | PPP6R1 | **73** | MOCS3 | **103** | SP2 | **133** | RCOR1 | **163** | GATAD2B |  |  |
| **14** | PTPN7 | **44** | LZTR1 | **74** | CMAS | **104** | MEN1 | **134** | CARM1 | **164** | ARPC2 |  |  |
| **15** | RPS6KA1 | **45** | GFI1 | **75** | GNPNAT1 | **105** | ARPC4 | **135** | SH2B3 | **165** | XRN1 |  |  |
| **16** | STUB1 | **46** | CEBPE | **76** | SPTLC2 | **106** | SRSF6 | **136** | HNRNPR | **166** | NIPBL |  |  |
| **17** | NCKAP1L | **47** | NF1 | **77** | DET1 | **107** | SASH3 | **137** | UBE2L3 | **167** | B4GALT1 |  |  |
| **18** | KAT6A | **48** | SPRED2 | **78** | SGMS1 | **108** | RBBP4 | **138** | ANAPC7 | **168** | ETV6 |  |  |
| **19** | PLEK | **49** | SLC38A2 | **79** | CREBBP | **109** | RPS6KA3 | **139** | RP11-45M22 | **169** | FLII |  |  |
| **20** | MEF2D | **50** | RPL28 | **80** | FBXO11 | **110** | C16ORF72 | **140** | MESDC2 | **170** | SPPL3 |  |  |
| **21** | PRKCB | **51** | AFF2 | **81** | SHOC2 | **111** | ARAF | **141** | KAT2A | **171** | AIP |  |  |
| **22** | TBL1XR1 | **52** | PRKD2 | **82** | NPRL2 | **112** | LAMTOR2 | **142** | ARPC3 | **172** | KIF23 |  |  |
| **23** | PPME1 | **53** | UBE2J1 | **83** | TM9SF3 | **113** | WASF2 | **143** | KRAS | **173** | FADD |  |  |
| **24** | UBE2D3 | **54** | BRK1 | **84** | JUNB | **114** | YPEL5 | **144** | JAK1 | **174** | LAMTOR4 |  |  |
| **25** | ZNF217 | **55** | FNDC3B | **85** | PIK3R5 | **115** | ZBTB2 | **145** | GNAI2 | **175** | C1GALT1C1 |  |  |
| **26** | XPR1 | **56** | RIC8A | **86** | ZDHHC5 | **116** | KAT7 | **146** | RRAGA | **176** | BASP1 |  |  |
| **27** | PRKCD | **57** | TNPO1 | **87** | AMBRA1 | **117** | POLD3 | **147** | ACTR2 | **177** | WDR81 |  |  |
| **28** | ERP44 | **58** | JKAMP | **88** | DEPDC5 | **118** | HIST2H2AA4 | **148** | GAB1 | **178** | BCL6 |  |  |
| **29** | USE1 | **59** | NANS | **89** | GET4 | **119** | CDK2 | **149** | ACTR3 | **179** | LAMTOR3 |  |  |
| **30** | CYFIP1 | **60** | SLC35A2 | **90** | FAM49B | **120** | STT3B | **150** | SUPT20H | **180** | FNIP2 |  |  |
